# Extent of pre-translational regulation for the control of nucleocytoplasmic protein localization

**DOI:** 10.1186/s12864-016-2854-4

**Published:** 2016-06-24

**Authors:** Mikael-Jonathan Luce, Anna Akuvi Akpawu, Daniel C. Tucunduva, Spencer Mason, Michelle S. Scott

**Affiliations:** Department of Biochemistry and RNA group, Faculty of Medicine and Health Sciences, University of Sherbrooke, Sherbrooke, Québec J1E 4 K8 Canada

**Keywords:** Protein targeting motifs, Nuclear localization signal, Nuclear export sequence, Alternative splicing, Pre-translational regulation, Protein subcellular localization, RNA-seq, Tissue-specific regulation

## Abstract

**Background:**

Appropriate protein subcellular localization is essential for proper cellular function. Central to the regulation of protein localization are protein targeting motifs, stretches of amino acids serving as guides for protein entry in a specific cellular compartment. While the use of protein targeting motifs is modulated in a post-translational manner, mainly by protein conformational changes and post-translational modifications, the presence of these motifs in proteins can also be regulated in a pre-translational manner. Here, we investigate the extent of pre-translational regulation of the main signals controlling nucleo-cytoplasmic traffic: the nuclear localization signal (NLS) and the nuclear export signal (NES).

**Results:**

Motif databases and manual curation of the literature allowed the identification of 175 experimentally validated NLSs and 120 experimentally validated NESs in human. Following mapping onto annotated transcripts, these motifs were found to be modular, most (73 % for NLS and 88 % for NES) being encoded entirely in only one exon. The presence of a majority of these motifs is regulated in an alternative manner at the transcript level (61 % for NLS and 72 % for NES) while the remaining motifs are present in all coding isoforms of their encoding gene. NLSs and NESs are pre-translationally regulated using four main mechanisms: alternative transcription/translation initiation, alternative translation termination, alternative splicing of the exon encoding the motif and frameshift, the first two being by far the most prevalent mechanisms. Quantitative analysis of the presence of these motifs using RNA-seq data indicates that inclusion of these motifs can be regulated in a tissue-specific and a combinatorial manner, can be altered in disease states in a directed way and that alternative inclusion of these motifs is often used by proteins with diverse interactors and roles in diverse pathways, such as kinases.

**Conclusions:**

The pre-translational regulation of the inclusion of protein targeting motifs is a prominent and tightly-regulated mechanism that adds another layer in the control of protein subcellular localization.

**Electronic supplementary material:**

The online version of this article (doi:10.1186/s12864-016-2854-4) contains supplementary material, which is available to authorized users.

## Background

Protein subcellular localization requires tight and timely regulation, to ensure proper environment and interaction partners, and ultimately function [1]. Localization regulation is achieved through diverse mechanisms which can act sequentially, combinatorially or competitively, the integration of which determines the localization distribution of proteins in the cell. In addition, protein localization is often dynamic, and mechanisms exist to allow translocation of proteins to respond to diverse changes in the cell and its environment.

Protein targeting motifs have been identified for all main eukaryotic cellular compartments and represent a highly prevalent mechanism regulating protein localization [2–5]. Targeting motifs typically involve short linear sequences of 3 to 30 amino acids, often found at protein ends or in accessible and/or disordered regions [6, 7]. The first targeting motifs that were described, over thirty years ago, were the signal peptide and the nuclear localization signal (NLS), specifying respectively entry into the secretory pathway through the endoplasmic reticulum, and targeting to the nucleus [8, 9]. In addition to targeting motifs, post-translational modifications (PTMs) are also often involved, either to modulate the accessibility of targeting motifs [10], to serve as a sorting signal [11, 12], or to anchor proteins in membranes by the addition of lipid chains [13, 14]. Other characterized mechanisms for the regulation of protein localization include targeting or more often retention through interactors which can include proteins, lipids and nucleic acid chains through the use of interaction domains [15–17]. Protein localization often results from the integration, in the proper order, of several of these mechanisms.

The regulation of translocation across the nuclear envelope has been particularly well characterized. Targeting to the nucleus from the cytoplasm typically involves NLSs, several classes of which have been described. Classical NLSs, the first to be identified, are short motifs involving basic residues, and can be divided into two main groups [18, 19]. Monopartite NLSs consist of a stretch of three to four basic residues [9, 18, 20] while bipartite NLSs are composed of two segments of basic residues separated by a linker of 10 to 12 residues [18]. Classical NLSs are recognized by Kapα-Kapβ1 importin heterodimers, of the karyopherin superfamily, for translocation across the nuclear pore complex and into the nucleus [19]. Many non-classical and more diverse NLSs have also been described, including combinations of polar/charged and non-polar residues [3, 21, 22]. More recently, longer nuclear targeting motifs recognized by the karyopherin Kapβ2 and averaging between 20 and 30 residues in length were described [23]. These PY-NLSs (Proline-Tyrosine Nuclear Localization Signals), unlike the classical NLS, do not have a strong consensus for their motifs, which are composed of a hydrophobic or basic N-terminal region and a C-terminal RX_2-5_PY motif [24].

Nuclear export sequences (NESs), specifying translocation from the nucleus to the cytoplasm have also been extensively characterized [25]. NESs are short motifs typically containing four hydrophobic residues, and most often leucines, separated by a small number of spacing residues [26]. NESs are also recognized by a member of the karyopherin superfamily of transport receptors, the CRM1 exportin, for export to the cytoplasm [25].

While the use of NLSs and NESs for nucleocytoplasmic transport is prevalent, some nuclear proteins do not contain these signals [20, 27]. Several such proteins employ other strategies to shuttle to and from the nucleus (for example by piggy-back onto other proteins that do contain NLSs [27–29]) but for most, targeting mechanisms are currently unknown [20]. NLSs and NESs are often regulated by PTMs, and their accessibility can also be regulated by conformational change, allowing a dynamic control of their usage [30, 31].

**Fig. 1:**
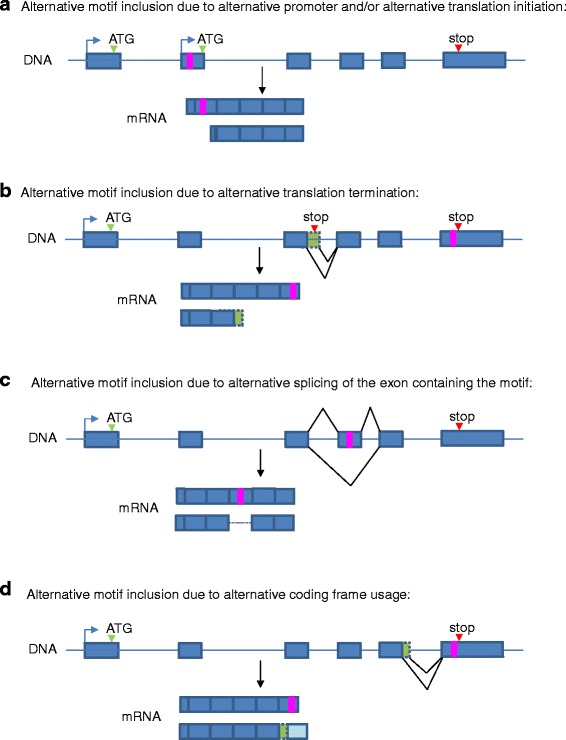
Modes of pre-translational regulation of the inclusion of protein targeting motifs. Four general mechanisms can regulate the inclusion of protein targeting motifs at the pre-translational level. The use of alternative promoters and/or alternative start codons can lead to different N-terminal protein ends differing in the presence of a targeting signal (**a**). Similarly, different C-terminal ends, which can be caused by alternative translation termination (**b**), can result in differential motif inclusion. Differential splicing of the exon containing the targeting motif (in this case, a cassette exon), can cause alternative motif inclusion (**c**). Alternative splicing can also result in a coding frameshift affecting the presence of a targeting motif (**d**). While alternative splicing can directly affect motif inclusion causing the motif to be spliced in or out (**c**), it can also indirectly regulate motif inclusion by affecting translation initiation (**a**) or termination (**b**), or by changing the coding frame (**d**). Start and stop codons are indicated respectively with green and red arrowheads, targeting motifs are shown as pink rectangles, exons are represented as boxes and introns as lines

In addition to the post-translational regulation of protein localization mentioned above, the targeting of proteins can also be regulated through pre-translational mechanisms, adding another level of complexity in the control of subcellular localization. In particular, the inclusion of targeting motifs in transcripts can be regulated by different types of pre-translational mechanisms. As illustrated in Fig. 1, alternative transcription/translation initiation sites, alternative splicing of the motif-encoding exon, alternative translation ends and coding frameshifts can all lead to protein isoforms encoded by the same gene but differing in the presence of targeting motifs [32–34]. Many different studies of individual genes have made light of such mechanisms which lead to the targeting of encoded proteins to more than one compartment. In particular, the subcellular distribution of many enzymes reflects the differential presence of mitochondrial targeting sequences or peroxisomal targeting sequences, as regulated at the pre-translational level [32–34]. On a transcriptome-wide level, the differential use of signal peptides and transmembrane domains as regulated at the pre-translational level has been investigated in mouse by considering all transcripts defined by the RIKEN FANTOM3 project [35, 36]. Similarly, the pre-translational regulation of short linear motifs, including short protein targeting motifs, has been characterized and classified by the Gibson group (for example, [37–39]). Collectively, these studies show that pre-translational regulation mechanisms represent an important and widely-used level of regulation of the inclusion of protein targeting motifs and ultimately of protein subcellular localization. However, the dynamic and diverse cellular roles of this type of regulation, have not been extensively and systematically investigated.

Here, we investigate of the extent of regulation of protein localization at the pre-translational level, through the study of targeting motif inclusion in transcripts, using the NLS and NES as model signals. The transcriptome-wide characterization of these targeting motifs reveals that 39 % of NLSs are constitutive in the sense that they are present in all coding isoforms of their encoding gene. The remaining 61 % of NLSs are considered to be alternatively regulated as they are not present in all coding transcripts of the same gene. In the case of NESs, 72 % are alternative. The inclusion of most alternative NLSs and NESs is regulated by alternative translation initiation and termination, although direct alternative splicing of the exon encoding the motif is also an important mechanism. The analysis of different deep-sequencing datasets in human indicates that the regulation of the inclusion of these targeting motifs at a pre-translational level can be dynamic and vary according to tissue-type, is more prominently used by proteins with diverse interactors, can be tightly regulated in a combinatorial way, and can be deregulated in disease states. Collectively, our findings show evidence of extensive and tightly-regulated use of pre-translational regulation mechanisms for the inclusion of the NLSs and NESs.

## Results

### Distribution of NLSs and NESs in transcripts/proteins

**Fig. 2:**
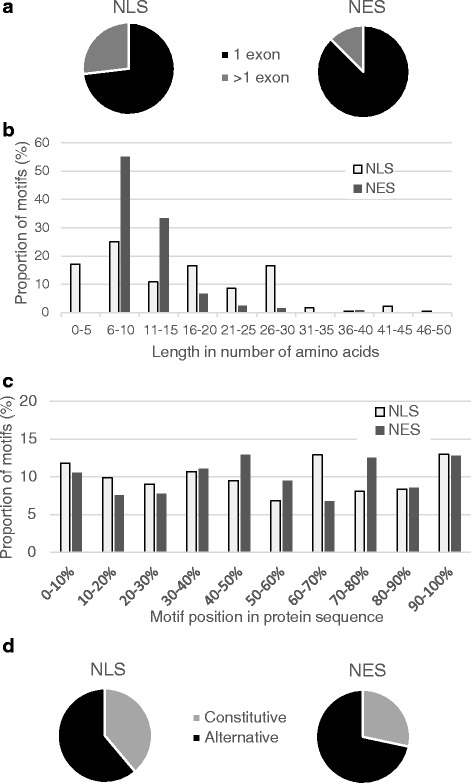
The positions of protein targeting motifs within genes and proteins are not uniformly distributed. **a** Most NLSs and NESs are entirely contained in only one exon. **b** Length distributions of NLSs and NESs. While NESs are represented by a unimodal length distribution, NLSs are characterized by a multimodal distribution, reflecting the diverse types of NLSs (monopartite, bipartite, PY-NLS) that have been described and that are considered in this study. **c** Distribution of the position of NLSs and NESs in proteins. **d** The majority of NLS and NES are regulated in an alternative manner

To characterize the extent of regulation of motif inclusion at a pre-translational level, we began by extensively curating the literature and public databases for experimentally validated human NLSs and NESs, as described in the Methods section. In doing so, we identified 175 NLSs present in 165 genes, and 120 NESs present in 102 genes as listed in Additional file 1. All NLSs and NESs were mapped onto the corresponding transcript and protein sequences and to the appropriate encoding exons using the hg38 assembly of the human genome and Ensembl annotations [40] as described in the Methods section. Analysis of the exonic position of these motifs reveals that 73 % of NLSs are entirely encoded in only one exon, while for NESs, the proportion goes up to 88 % (Fig. 2a). The difference between NLSs and NESs can be explained in part by the fact that NLSs are on average longer than NESs, making them more likely to be encoded on more than one exon (Fig. 2b). However, when compared to randomly chosen protein subsequences of the same length distribution as the respective motifs, significantly more NESs, but not NLSs, are entirely encoded in one exon than expected by chance (*p*-value = 0.03 for NES vs 0.9 for NLS), suggesting that NESs are under pressure to remain modular in terms of alternative inclusion potential. In addition, some of these motifs are present on small exons that seem to have appeared solely for the conditional inclusion of the motif, as discussed with specific examples in following sections.

The position of NLSs and NESs in protein sequences might also influence how the motif is regulated at a pre-translational level. As shown in Fig. 2c, NLSs and NESs can be present throughout the protein and do not have strong preferences for protein ends. Positioning of motifs in the protein will influence the modes of regulation used to control motif inclusion. To investigate this, we set out to classify and characterize the prevalence of mechanisms regulating the presence of these motifs in transcripts from all genes containing them.

### Alternative regulation of the inclusion of NLSs and NESs

**Fig. 3:**
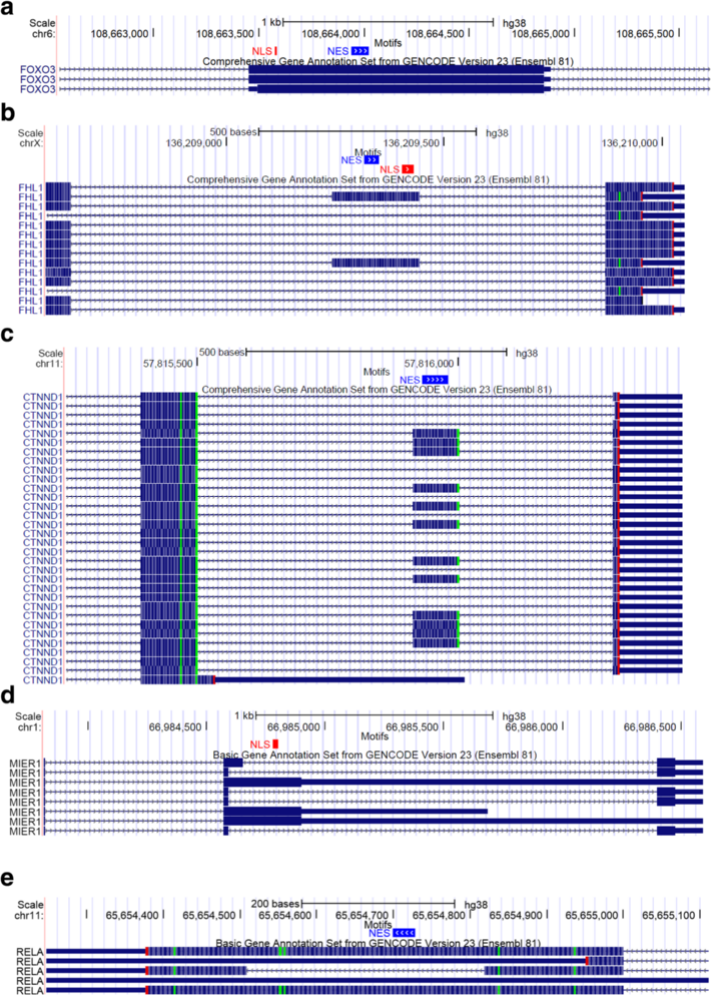
Examples of constitutive and alternative splicing-regulated NLSs and NESs. Screenshots of the UCSC Genome Browser displaying portions of transcripts encoding protein targeting motifs. NLSs and NESs are represented by red and blue blocks respectively, in the Motifs track. The Comprehensive Gene Annotation Set from GENCODE Version 23 (Ensembl 81) track shows the position of exons (blocks) and introns (lines with arrows showing direction of transcription) in the chromosome window considered. Coding exons are represented by thick blocks whereas non-coding exons (UTRs or non-coding transcripts) are represented by thin blocks. **a** Constitutive NLS and NES in FOXO3 gene. **b** FHL1 cassette exon containing NES and NLS. **c** Cassette exon of CTNND1 gene, containing NES. **d** MIER1 NLS encoded in exon regulated by alternative 5′ splice site. **e** RELA NES encoded in portion of exon that can be spliced out (intronic exon)

To investigate the distribution of the presence of motifs for all genes containing NLSs and NESs, we considered all coding transcripts defined in the Ensembl database [40] for assembly hg38 of the human genome, as described in the Methods. In total, 39 % (68/175) of NLSs and 28 % (34/120) of NESs are present in all coding transcripts of their encoding gene, and are referred to as constitutive motifs (Fig. 2d). The remaining motifs are alternative in the sense that not all coding transcripts of the encoding genes contain the motif. An example of constitutive motifs is shown in Fig. 3a: the FOXO3 NLS and NES are both contained in an exon present in all 3 transcripts of the gene.

**Fig. 4:**
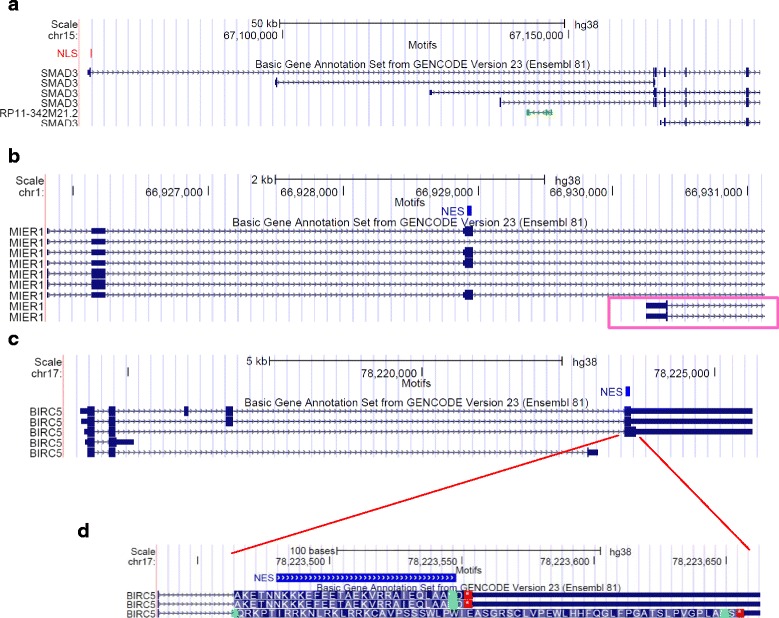
Examples of alternative NLSs and NESs regulated by alternative transcription/translation initiation or termination. Screenshots of UCSC Genome Browser as described in Fig. 3. **a** SMAD3 NLS regulated by alternative transcription and alternative initiation. **b** The MIER1 NES is encoded in a cassette exon and its inclusion is also regulated by alternative transcription and alternative initiation (some transcripts, highlighted in a pink box, start at a downstream exon). **c** The BIRC5 NES inclusion is regulated by alternative transcription and translation end (some transcripts end before the exon containing the targeting motif) and by a frameshift (visible in panel **d**, which represents a zoomed-in section of panel **c**)

Of the modes of pre-translational differential regulation of targeting motif inclusion previously observed [32–34] and summarized in Fig. 1, examples of each type were identified in the regulation of NLSs and NESs. While cassette exons are the most common type of alternative splicing mechanisms regulating exons containing NLSs and NESs, we also observe alternative 5′/3′ splice sites and an exonic intron as shown in Fig. 3 b-e. However, the most prevalent mechanisms of alternative regulation of NLS and NES inclusion are the alternative translation initiation and termination sites, which result in proteins with different N-termini and C-termini respectively, as shown in Fig. 4. For example, 5 isoforms of the SMAD3 gene are defined in the Ensembl annotations, all differing in their N-termini due to the usage of different promoters, and as a consequence, different start codons. Only the longest isoform has the NLS, right at its N-terminal extremity (Fig. 4a). Similarly, the presence of the MIER1 NES is alternatively regulated as several coding transcripts start downstream of the exon encoding the motif. The MIER1 NES is an example of a motif regulated by several pre-translational mechanisms as this motif is encoded in a cassette exon and is thus regulated by both alternative splicing and alternative transcription/translation initiation (Fig.4b). The presence of the BIRC5 NES is also regulated by more than one mechanism as not only are there two coding transcripts that end before the beginning of the motif, but alternative splicing of upstream exons causes a frameshift of the exon encoding the NES in one coding transcript (Fig. 4c,d). We note that alternative translation initiations and terminations, as well as frameshifts, are often indirectly caused by alternative splicing of upstream exons (see for example some transcripts in Fig. 4c, d). Thus alternative splicing, whether directly of the exon encoding the motif, or of exons further upstream, is a central mechanism in the pre-translational regulation of protein targeting motif inclusion. The regulation modes found for each motif are indicated in Additional file 2 for NLSs and Additional file 3 for NESs.

**Fig. 5:**
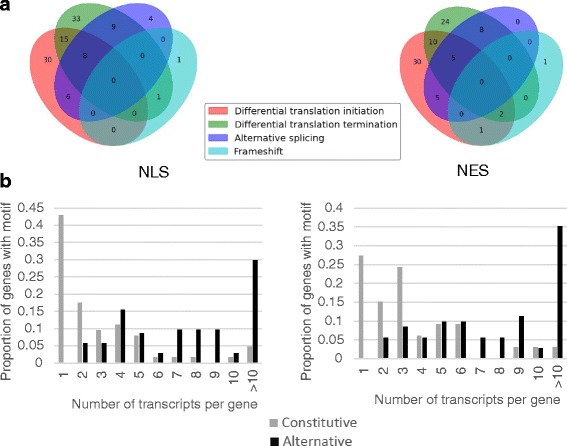
Number of motifs regulated by the different pre-translational regulation modes. Of the 175 NLSs considered, 68 are constitutive. The 107 alternatively regulated NLSs are classified with respect to 4 modes of regulation in panel **a** (left): direct alternative splicing of the encoding exon, differential translation initiation, differential translation termination and frameshift. Similarly, of the 120 NESs considered, 34 are constitutive and panel **a** (right) shows the regulation types of the 86 alternative NESs. Alternatively regulated NLSs and NESs are present in genes producing significantly more transcripts than constitutive NLSs and NESs as shown in **b** (NLS on the left panel and NES on the right)

To determine the extent of usage of the different pre-translational regulation mechanisms observed and defined in Fig. 1, we classified all NLSs and NESs considered (listed in Additional file 1) according to their regulation modes (as shown in Fig. 5a). While the inclusion of 25 % and 21 % of alternative NLSs and NESs respectively are directly regulated by alternative splicing, a much larger proportion, in fact almost all alternative NLSs (95 %) and alternative NESs (99 %) are regulated by alternative initiation and/or alternative end. Alternative initiation and termination were previously found to be predominant regulatory mechanisms for the inclusion of signal peptides and transmembrane domains [35, 36]. A subset of alternative NLSs and NESs (36 % for both NLSs and NESs) are regulated by more than one mechanism (for example, Fig. 4b-d). Thus the pre-translational regulation of NLS and NES inclusion is widespread and uses several distinct mechanisms, often tightly regulating the inclusion of the motif with little extra flanking sequence (for example Figs. 3c, 4).

We investigated the distribution of the number of coding transcripts containing an NLS or NES constitutively or alternatively. Interestingly, alternative NLSs and NESs are found in genes encoding significantly more transcripts than those containing constitutive NLSs and NESs (Fig. 5b, *p*-value < 6.3*10^−6^ for NES and *p*-value < 5.2*10^−12^ for NLS using the two sample Kolmogorov-Smirnov test) indicating that genes containing such alternative motifs encode on average a larger and more diverse set of proteins than genes containing constitutive motifs and that the regulation of motif inclusion is responsible for some of the need for co- and post-transcriptional regulation, as previously shown for signal peptides and transmembrane domains [35, 36].

When NLSs are classified according to their subtype as described in the Methods, PY-NLSs are found to be the most regulated in an alternative manner and most likely to be present in more than one exon (67 % of PY-NLSs are alternative and 38 % are encoded in 2 exons; Additional file 4: Figure S1). Unlike monopartite NLSs, bipartite NLSs and non-classified NLSs which are two to four times more likely to be regulated by alternative translation initiation and termination than by splicing, PY-NLSs display equal counts for these three types of regulation (Additional file 4: Figure S1). Thus the diverse group of PY-NLS stands out as the most alternatively regulated subgroup of NLSs.

### Quantitative analysis of motif inclusion across normal human tissues

**Fig. 6:**
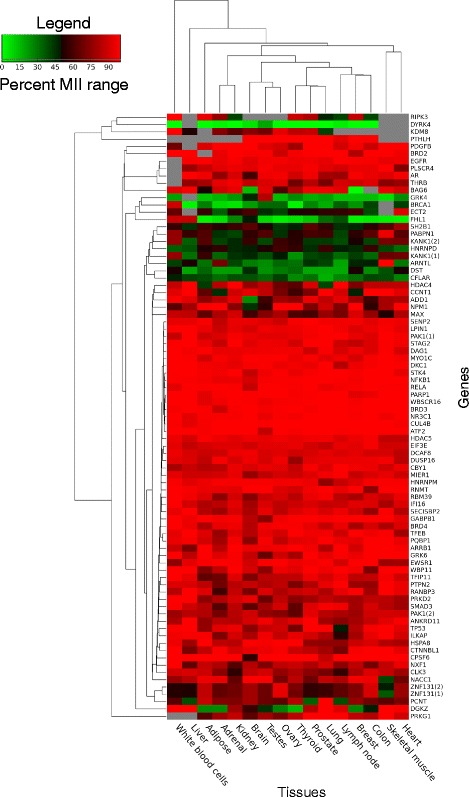
Heatmap representing the level of motif inclusion for alternative NLSs. The Human Body map project RNA-seq data was used to calculate MII values for NLS motifs in each tissue considered. Only alternatively regulated motifs present in at least 9 of the 16 tissues considered are represented here. MII values are represented using the color scheme depicted in the legend. Genes which were detected below a threshold of 1 TPM are represented by grey cells in the heatmap

**Fig. 7:**
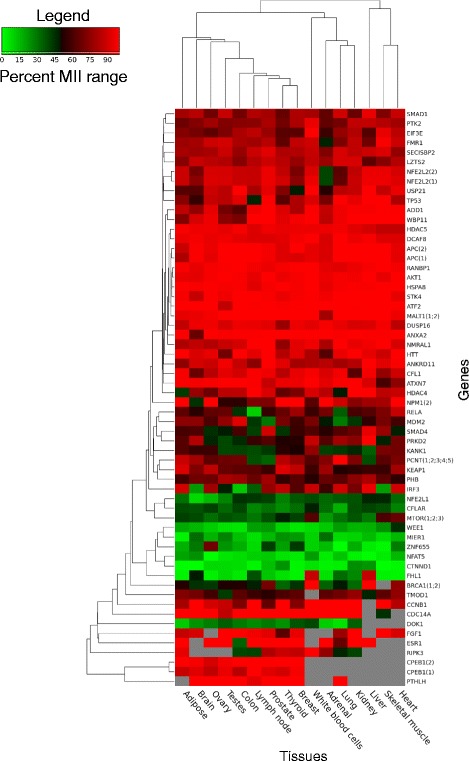
Heatmap representing the level of motif inclusion for alternative NESs. As for Fig. 6, the Human Body map project RNA-seq data was used to calculate MII values for NES motifs in each tissue considered. Only alternatively regulated motifs present in at least 9 of the 16 tissues considered are represented here. Motifs of same type present in the same gene and displaying the exact same MII profile across all tissues were collapsed into one entry (for example, BRCA1 has two annotated NESs with the same MII profile across all tissues. These motifs were collapsed into one entry labelled BRCA1(1;2))

The above analysis considers all coding transcripts defined in the Ensembl database for a given gene. However, the relative cellular abundance of the transcripts with the motif compared to the transcripts without the motif is undefined in the above analysis. We note that for a gene containing an alternatively regulated motif as defined above, if all its transcripts lacking the motif are expressed at very low level, such an ‘alternative’ motif will in fact behave like a constitutive motif when quantified and when used in the cell. To estimate the true level of motif inclusion in transcriptomes, we analysed RNA-seq data from the Illumina Human Body Map project (NCBI GEO accession GSE30611), which provides data for 16 normal human tissues. As described in the Methods section, to quantify motif inclusion, we define the motif inclusion index (MII), which represents the relative abundance of all coding transcripts containing the motif out of all coding transcripts from the gene. The MII thus ranges between 0 and 1, 0 representing motifs for which out of all coding transcripts produced by the encoding gene, only those not containing the motif are detected, while an MII of 1 is calculated for motifs for which all coding transcripts produced by the encoding gene contain the motif. Out of the 165 and 102 genes containing NLSs and NESs respectively according to our above analysis of the transcripts from the Ensembl database, 142 and 91 of these genes were detected above a set cut-off (total abundance of the gene of at least 1 transcript per million (TPM) in at least 9 of the 16 tissues considered) and the other genes were not further considered. Unsurprisingly, all NLSs and NESs classified above as constitutive obtain MII values of 1. MII of both alternative NLS and alternative NES cover the whole range between 0 and 1. Approximately 10 % of NLSs and NESs (15 NLSs and 8 NESs) that we classified as alternative according to the Ensembl transcripts definitions obtain MII values above 0.95 for all human tissues with RNA-seq data available in this dataset, and were considered to be regulated like constitutive motifs. While we cannot exclude that these motifs might be alternatively included in transcripts in other tissue types and/or conditions not sampled, we currently have no evidence of their alternative regulation at appreciable levels. The inclusion indexes of the remaining NLSs and NESs with MII values below 0.95 in at least one tissue (alternative motifs as defined by RNA-seq analysis) are shown in Figs. 6 and 7. For both alternative NESs and alternative NLSs, a small subset of genes have either uniformly high MII or uniformly low MII, but the majority have variable MII values across tissues, showing tissue-specificity in the regulation of the inclusion of these motifs. For example, the PRKD2 NES MII goes from around 0.40 in testes to above 0.80 in brain and white blood cells and as high as 1.0 in liver tissue (Fig. 7), while the BAG6 NLS MII ranges from below 0.15 in breast and brain tissue to above 0.90 in the liver, lung, testes, prostate, kidney and lymph nodes (Fig. 6). PRKD2 (protein kinase D2) is a member of the PKD family of serine/threonine kinases that mediate signals from diverse pathways including T-cell receptor, G-protein-coupled receptor and MAPK signaling pathways and can activate the NF-kB pathway following stress signals [41–43]. PRKD2 is found both in the nucleus and the cytoplasm [42, 44] and has many interactors and substrates (for example [41–43]). In the nervous system, PRKD2 plays an essential role in the establishment and maintenance of neuronal polarity through activity at the Golgi apparatus [45, 46], supporting the need for a high NES MII value in brain. The differential inclusion of the PRKD2 NES thus likely reflects its diverse cellular functions and interactors in different tissues. Similarly, BAG6 is also found in both the nucleus and cytoplasm and is involved in diverse functions in both these compartments, including serving as a chaperone for the insertion of tail-anchored proteins at the endoplasmic reticulum and playing a role in DNA damage induced apoptosis in the nucleus through the acetylation of p53 [47–49]. In testes, BAG6 has been characterized as a nuclear protein involved in the regulation of chromatin structure and gene expression through the recruitment of histone modifiers [50], supporting the high NLS MII found for BAG6 in testes. In contrast, other studies find BAG6 mainly in the cytoplasm, playing a role in the proteasomal degradation of misfolded proteins during endoplasmic reticulum-associated degradation (ERAD) by maintaining polypeptides in soluble states [51, 52]. As in the case of PRKD2, the regulation of the targeting motif inclusion of BAG6 likely reflects the distribution of its numerous interactors and functions in the different tissue types. In general, the genes with alternative motifs displaying a wide range of MII values across tissues have many interactors and/or annotated functions, many being kinases (10 with alternative NLS, 8 with alternative NES) or phosphatases (4 with alternative NLS, 2 with alternative NES).

### Co-regulation of NLS and NES

**Fig. 8:**
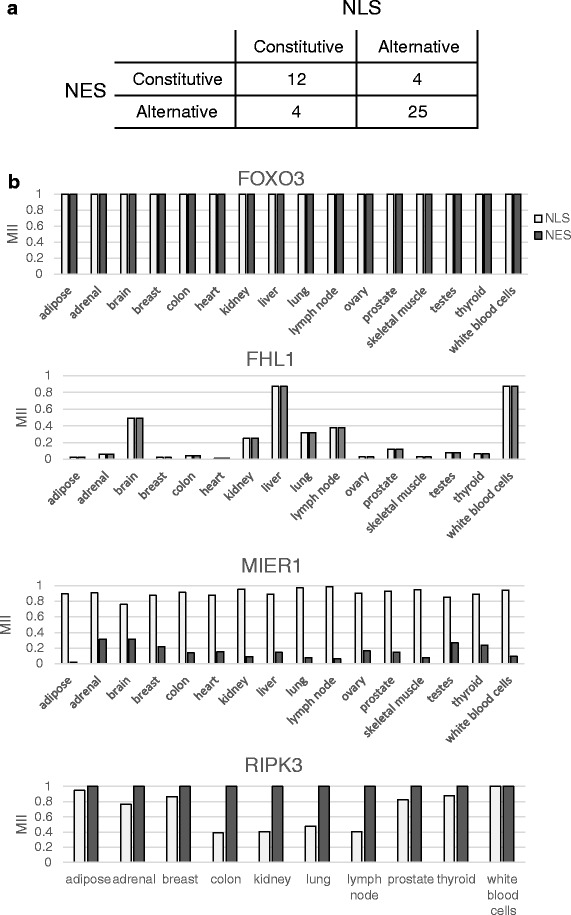
Co-regulation of NLS and NES. 45 genes encoding both an NLS and NES were detected by RNA-seq in the Human Body Map project. The distribution of their regulation types are shown in panel **a**, with both motifs being constitutive or both being alternative as the most abundant groups. The distribution of motif inclusion for both the NLS and NES in a subset of these genes is shown in panel **b**. FOXO3 represents a gene with both constitutive NLS and constitutive NES. FHL1 represents the group of genes with co-regulated but alternative NLS and NES. MIER1 and RIPK3 have differentially regulated NLS and NES, with MIER1 showing high prevalence of NLS and low presence of NES while RIPK3 has a constitutive NES and an alternative and variably present NLS

Of the 45 genes encoding both an NLS and an NES and detected in the Human Body Map project, the majority (>75 %) are annotated as nuclear and cytoplasmic according to Uniprot, several shuttling between these compartments. As described in Fig. 8a, of these genes, 27 % (12/45) have both a constitutive NLS and a constitutive NES (for example FOXO3 in Fig. 8b) while 56 % (25/45) have an NLS and an NES that are both alternative (for example FHL1 and MIER1 in Fig. 8b), showing concordance in the pre-translational regulation of these motifs (*p*-value = 7.0*10^−5^ by Fisher’s exact test). Amongst the 25 genes with both an alternative NLS and alternative NES, 32 % (8/25) show complete co-regulation of their alternative NLS and NES, encoding the motifs in the same or co-regulated exons. For example, although highly variable between tissues (MII going from to 0.016 in the heart to 0.88 in white blood cells for both the NLS and NES), in any given tissue, the MIIs of FHL1 are the same for its NLS and its NES (Fig. 8b). FHL1 (Four and a Half LIM domains protein 1), also referred to as SLIM1 [53], is an ion channel binding protein involved in cell differentiation and organ morphogenesis, primarily found in the cytoplasm and particularly, at focal adhesions at the plasma membrane according to Uniprot [47] and the HPA [44]. Both its NLS and NES are encoded in a cassette exon which is only included in two of the 14 coding transcripts produced by the gene (Fig. 3b). By co-regulating its NLS and NES, such a gene ensures that the encoded proteins will either be solely cytoplasmic (absence of both the NLS and NES, which is most often the case for FHL1), or capable of localizing to both the cytoplasm and nucleus, and cycling between them. A little under half of genes encoding both an NLS and NES ensure the complete co-occurrence of these motifs in their transcripts across the 16 human tissues considered.

Of the remaining 56 % of genes containing both an NLS and NES, but with less or no coordinated occurrence of the motifs (for example MIER1 and RIPK3 in Fig. 8b), the majority show a preference for one of the two motifs across all tissues. For example, the MIER1 NLS is much more prevalent than its NES, while in RIPK3, the NES is always present but the NLS can have a MII as low as 0.39. MIER1 (mesoderm induction early response protein 1) proteins are transcriptional corepressors known to function in the nucleus, although some have been detected in the cytoplasm [54]. Alternative splicing and alternative translation initiation sites result in proteins differing in the presence of their NLS and/or NES [55] (as shown in Figs. 3d, 4b). The uniformly high NLS MII and low NES MII that we observe (Fig. 8b) are consistent the mainly nuclear role of the protein in normal tissues. In contrast, although known to be capable of translocating to the nucleus during necroptosis [56], RIPK3 is annotated as mainly functioning in the cytoplasm, propagating the signal from the tumor necrosis factor receptor by phosphorylating its substrates [57, 58], consistent with the presence of a constitutive NES and an alternative NLS. In general, many of the genes encoding both an NLS and NES that are not co-regulated encode proteins that have either large numbers of interactors and diverse functions, including for example PRKD2, SENP2 and KANK1, or have many isoforms annotated as localized in diverse and different compartments (for example MIER1 and PRKD2). Most of these regulate the presence of these motifs in a tissue-specific manner, some displaying switches between a strong presence of the motif (MII near 1) and a near absence (MII near 0) of one of their motifs between different tissues. A small number of patterns of motif inclusion are predominantly used by the cell, and represent tightly controlled programs.

### Quantitative analysis of motif inclusion across a panel of breast cancer tissues

As done for the Human body map RNA-seq datasets, the motif inclusion of NLS and NES was quantified across a panel of breast cancer datasets comparing estrogen-positive tumors (ER+), triple negative tumors, HER2-positive tumors (HER2+) and benign tumors [59] (Additional file 4: Figures S2–S3). Once again, amongst the alternative motifs, a subset of genes display strongly included or strongly excluded NLSs and NESs, with high overlap and same general distribution with the equivalent subsets in the Human Body Map datasets. Despite these general trends, cancer type specific patterns also emerge. For example, the benign breast cancer samples generally cluster separately from the ER+, triple negative and HER2+ breast tumors, in particular for the NLS heatmap, when looking at genes displaying variable MII values, indicating that the inclusion of a subset of these alternative motifs is differentially regulated between benign cell lines and tumors. Such genes include CPSF6, PABPN1, ARNTL, KANK1 and DST, which show striking differences in the NLS MII when benign and non-benign samples are compared (Additional file 4: Figure S2). While some of these genes have been described as either strongly mutated, deleted, deregulated or involved in pathways that are deregulated in specific types of breast-cancer [60, 61], their potential deregulation of localization has not been investigated. These results suggest that specific changes in the inclusion of protein targeting motifs, as regulated at pre-translational levels, might represent events specific to certain tumor types, and could be used as novel biomarkers. They might contribute to cancer phenotype and their study could lead to insight into cancer maintenance and progression.

## Discussion and Conclusions

Timely regulation of protein subcellular localization is crucial and underlies many cellular pathways. While protein localization can be controlled through several post-translational mechanisms, cells also regulate protein localization by varying the inclusion of targeting motifs at pre-translational levels [32, 33, 37, 38]. Here, we describe the extensive cellular use of these mechanisms for the control of nucleo-cytoplasmic traffic through the study of the inclusion of NLSs and NESs. The analysis of experimentally validated human NLSs and NESs indicates that these motifs are modular and that their inclusion is regulated by the use of alternative promoter and/or translation initiation, as previously described for signal peptides [35, 36], as well as by alternative splicing, by alternative translation termination, and also by coding frameshift for a small number of genes. Alternative initiation and termination are the predominant mechanisms in use for this regulation as was found for signal peptides and transmembrane domains [36]. The inclusion levels of these motifs, as analyzed quantitatively using RNA-seq datasets, vary from 0 to 100 %, depending on the gene and the tissue type. While many NLSs and NESs are highly included (most or all transcripts generated from the gene containing the motif), others are included at very low levels or at variable levels which, for well characterized proteins, can be explained by their molecular function. A majority of these motifs are not present in a constitutive manner (61 % of NLSs and 72 % of NESs are alternative) making the pre-translational regulation of the inclusion of these motifs a widely used mechanism in the regulation of protein cellular localization.

The pre-translational regulation of the inclusion of targeting motifs is the first of several levels of regulation for these localization signals. Subsequently, once included in proteins, the accessibility of targeting motifs can be modulated by interaction with other molecules or by allostery, and can also be regulated by post-translational modification [37]. In addition, the presence of different targeting motifs within the same protein can lead to competition between the motifs to determine the final localization. These distinct levels of regulation serve different purposes and exhibit different characteristics. While the regulation of targeting motif accessibility is typically a reversible regulation, the pre-translational regulation of their inclusion is irreversible [37, 39], and thus the cell commits to the level of motif inclusion it chooses, and has less flexibility for immediate responses requiring localization translocation. Nonetheless, this mode of regulation does provide the possibility of co-regulation in the case of proteins with significantly different sets of interactors depending on their localization, as seems to be the case in particular for some kinases shuttling between the nucleus and cytoplasm. Thus the inclusion of specific targeting motifs could be coordinated to occur when their substrates/interactors present in the targeted compartment are expressed, for example. The further characterization of this widespread mechanism of regulation of protein localization and the study of its use in combination with post-translational regulation mechanisms will shed light on and lead to better models of the regulation of this fundamental protein characteristic and the causes of its deregulation in disease states.

## Methods

### Data collation

Human NLSs and NESs were obtained from specialized databases and by manual curation of the literature. 58 NLSs were obtained from the database of experimentally validated localization signals LocSigDB [62] including 19 NLSs that are also present in NLSdb [63]. Many additional NLSs were identified by manual curation of the literature including 24 PY-NLSs described and listed in [24]. To be included in the list, we required experimental validation including deletion/mutation analysis and targeting of reporter proteins to the nucleus. 116 NESs were obtained from the database of validated NESs ValidNES [64] and 4 additional NESs by literature curation. References for all NLSs and NESs considered are available in Additional file 1 as well as information regarding the database from which they were extracted and a reference to the article in which their validation is described. NLSs and NESs were only kept if they could be mapped onto their corresponding encoding protein and if their reported amino acid sequence did not exceed 50 amino acids in length [7], to ensure we are not working with signal patches.

### Motif position analysis in exons and proteins

Transcripts and protein sequences, and their genomic positions as well as exon positions were obtained from the Ensembl database human genome build hg38, version 82 [40]. No patches were applied. All data was managed in an in house MySQL database. Motif sequences were mapped onto the encoding protein and then onto the corresponding transcript and ultimately onto the corresponding exon(s), by considering the position of the start codon (coding start) and the positions of all exons obtained from the Ensembl annotations [40], allowing the evaluation of the number of exons in which the motif is present. A sampling procedure randomly choosing the same number of subsequences of same length as NLSs or NESs from all proteins defined in hg38 was used to evaluate the random distributions.

For the distribution of NLSs and NESs in protein sequences, the position of the first residue of the motif was identified in the corresponding protein. The relative position in the protein was obtained using the following formula:

$$ \mathrm{Relative}\ \mathrm{motif}\ \mathrm{position} = {\mathrm{P}}_{\mathrm{m}}/\ \left({\mathrm{L}}_{\mathrm{p}}\hbox{-} {\mathrm{L}}_{\mathrm{m}}\right) $$

where P_m_ is the position of the first residue of the motif in the protein, L_p_ is the length of the protein and L_m_ is the length of the motif.

The relative motif positions were then binned and the resulting counts represented as histograms. To ensure equal representation of genes regardless of the number of isoforms encoded, each gene was given an equal weight in the counts. As genes can code for different isoforms that do not all encode the motif at the same position in the resulting proteins, each coding isoform encoding the motif was considered and given a partial count for the gene, the total count for the gene totaling 1.

### Classification of NLSs

NLSs were classified according to their type using the following criteria:

- Bipartite NLSs were defined as those matching the PDOC00015 prosite profile (two adjacent basic amino acids (Arg or Lys), a spacer region of any 10 residues, at least three basic residues (Arg or Lys) in the five positions after the spacer region) [65].
- Monopartite NLSs were required to conform to the consensus sequence K(K/R)*X*(K/R) defined in [20].
- PY-NLSs were defined in the paper [24].
- All remaining NLSs were annotated as non-classified with respect to their subtype.

### Mode of pre-translational regulation of motif inclusion

A custom track specifying the positions of all NLSs and NESs was generated for visualization with the UCSC Genome Browser by considering the relative position of the motif in the protein sequence, the absolute position of the coding start of the transcript in the hg38 genome build and the absolute positions of the exons of the transcripts in the hg38 genome build. Constitutive motifs were defined as motifs present in all coding transcripts of a gene. In contrast, motifs are considered alternative if there exists at least one coding transcript of the encoding gene that does not contain the motif. Motifs were classified according to the types of pre-translational regulation modulating their inclusion (as defined in Fig. 1) by considering all coding transcripts of the encoding genes using in house scripts. Motifs are considered absent from a transcript if their sequence (according to Additional file 1) is not entirely included in an isoform.

### Quantification of motif inclusion by RNA-seq

To quantitatively determine the relative abundance of the transcripts containing the motif compared to the other transcripts of the same gene, we analyzed high-throughput sequencing datasets of 16 different normal human tissues from the Illumina Human Body Map Project (NCBI GEO accession GSE30611). The RNA-seq datasets for the 16 tissues consisted of between 74 and 82 million paired-end reads. The sra-toolkit was used to extract the fastq files from the sra archived datasets [66] using the fastq-dump command with split-files option. Reads were aligned to the hg38 assembly of the human genome, and quantified per transcript using Kallisto, with the command line kallisto index –k21 [67].

The proportion of transcripts containing a motif of interest out of all transcripts produced from a gene is referred to as the motif inclusion index (MII):

$$ MII\left(g,\ m\right) = \frac{{\displaystyle {\sum}_{k\ \in K}}{A}_k}{{\displaystyle {\sum}_{n\ \in N}}{A}_n} $$

where N represents the set of all coding transcripts encoded by gene g, K represents the set of all coding transcripts encoded by gene g and containing motif m (K ⊆ N) and A represents a transcript’s relative abundance in TPM. The MII values were only calculated for genes with a total abundance of above 1 TPM for a given dataset.

The GSE45419 datasets consisting of benign breast lesions, ER positive, triple negative and HER2 positive primary breast tumors [59] were analyzed in the same way as the Human Body Map Project datasets as described above.

## Additional files

Additional file 1: Experimentally validated NLS and NES motifs considered in this study, with their sequence and reference. (PDF 341 kb) Additional file 2: Classification of all NLS motifs considered in this study, including their sequence, class, regulation modes, number of encoding transcripts and number of encoding exons. (XLSX 23 kb) Additional file 3: Classification of all NES motifs considered in this study, including their sequence, regulation modes, number of encoding transcripts and number of encoding exons. (XLSX 15 kb) Additional file 4:
**Figure S1.** Characteristics of NLS subtypes. NLSs were classified according to their type as described in the Methods. The number of NLSs of different subtype with the indicated characteristics are shown. **Figure S2.** Heatmap representing the level of motif inclusion for alternative NLSs. The GSE45419 RNA-seq datasets consisting of benign breast lesions, ER positive, triple negative and HER2 positive primary breast tumors (32 samples in total) were analyzed to quantify the NLS MII in each tissue considered. MII values are represented using the color scheme depicted in the legend. Genes which were detected below a threshold of 1 TPM were not further considered and are represented by grey cells in the heatmap. Only motifs present in at least 17 of the 32 samples are shown. The sample type is indicated using a color-coded bar at the top of the heatmap. While some NLSs display generally uniform MII values across the samples regardless of type, a subset show specificity for a certain sample type. In particular, the benign breast lesions cluster together and display different MII values for some NLSs, suggesting a differential regulation of the presence of the NLS in these samples. **Figure S3.** Heatmap representing the level of motif inclusion for alternative NESs. As for figure S2, the GSE45419 RNA-seq datasets were analyzed to quantify the NES MII in each tissue considered. The heatmap is as described for figure S2. Motifs of same type present in the same gene and displaying the exact same MII profile across all tissues were collapsed into one entry (for example, CDC7 has two annotated NESs with the same MII profile across all tissues. These motifs were collapsed into one entry labelled CDC7(1;2)). (PDF 419 kb)
