# Supplementary material for: Extent of pre-translational regulation for the control of nucleocytoplasmic protein localization
Source: BMC Genomics. 2016 Jun 24;17:472. doi: 10.1186/s12864-016-2854-4 (PMC4919871; doi:10.1186/s12864-016-2854-4)
Supplement: Additional file 1: — Experimentally validated NLS and NES motifs considered in this study, with their sequence and reference. (PDF 341 kb) [file 12864_2016_2854_MOESM1_ESM.pdf]

Additional Table 1: Experimentally validated NLS and NES motifs considered in this study, with their sequence and reference.

Gene name	Signal type[a]	Sequence	Reference	Database[b] supporting annotation
ACD	NES	LVCLAESCLTLE	[1]	ValidNES
ADARB2	NLS	KRRRRRRSKRKDK	[2]	
ADD1	NLS	KKKKKFRTPSFLKKSKK	[3]	
ADD1	NES	LMRMLDNL	[3]	
AHR	NES	KLDKLSVLRLS	[4]	ValideNES
AHR	NLS	RKRRKPVQKTVKPIPAEGIKSNPSKRH	[4]	
AICDA	NES	LRDAFRTLGL	[5]	ValidNES
AICDA	NLS	RRKFLYQFKNVRWAKGRR	[5]	
AKT1	NES	DLKLENLMLDK	[6]	ValidNES
ANKRD11	NES	LAAIVDAIKL	[7]	ValidNES
ANKRD11	NLS	RKRIKK	[7]	
ANP32B	NES	LKKLECLKSLDL	[8]	ValidNES
ANP32B	NLS	KRKRE	[8]	
ANXA2	NES	MSTVHEILCKLSLEGDHSTPPSAYGSVKA	[9]	ValidNES
APC	NES (1)	LTKRIDSLPL	[10]	ValidNES
APC	NES (2)	LLERLKELNL	[10]	ValidNES
APLNR	NLS	RKRRR	[11]	
AR	NLS	RKCYEAGMTLGARKLKK	[12]	LocSigDB
ARNT	NLS	RAIKRRPGLDFDDDGEGNSKFLR	[13]	LocSigDB, NLSdb
ARNTL	NLS	RKRKG	[14]	
ARRB1	NLS	KIHKRNSVRLVIRK	[15]	

ATF2	NLS	RRRRAANEDPDEKRRKFLERNRAAASRCRQKR	[16]	
ATF2	NES	VAQLKQLLL	[16]	ValidNES
ATF3	NLS	RKKRRRERNKIAAAKCRNKKKE	[17]	
ATXN7	NES	FDPDIHCGVIDL	[18]	ValidNES
BAG6	NLS	RKVKPQPPLSDAYLSGMPAKRRK	[19]	
BIRC5	NES (1)	VKKQFEELTL	[20]	ValidNES
BIRC5	NES (2)	NKKKEFEETAEKVRRAIEQLAAM	[20]	ValidNES
BLM	NLS	RKRKKMPASQRSKRRK	[21]	LocSigDB, NLSdb
BOK	NES	LLRLGDELE	[22]	ValidNES
BRCA1	NES (1)	LECPICLEL	[23, 24]	ValidNES
BRCA1	NES (2)	QLVEELLKIICAFQLDTGL	[24]	ValidNES
BRCA1	NLS	PKKNRLRR	[25]	LocSigDB, NLSdb
BRCA2	NES	DLSDLTFLEVA	[26]	ValidNES
BRCA2	NLS (1)	KKRR	[27]	LocSigDB
BRCA2	NLS (2)	PIKKK	[27]	LocSigDB
BRD2	NLS	KKGVKRKADTTTP	[28]	
BRD3	NLS	KKGVKRKADTTTP	[28]	
BRD4	NLS	KKGVKRKADTTTP	[28]	
BRMS1	NLS	PPSKRKK	[29-31]	
CBY1	NES	LSNLHSLDR	[32]	ValidNES
CBY1	NLS	RKRK	[32]	
CCNB1	NES	DLCQAFSDVILA	[33]	ValidNES
CCNT1	NLS	VNMLHSLLSAQGVQPTQPTAFEFVRPY	[34]	
CDC14A	NES	ILSGLDDMSI	[35]	ValidNES
CDC14B	NES	LLSGVDDISI	[35]	ValidNES
CDC7	NES	LLDKLLDLNP	[36]	ValidNES

CDC7	NES	LRKLCERLRG	[36]	ValidNES
CDKN1A	NES (1)	LQGTAEEDHVDLSLSCTL	[37]	ValidNES
CDKN1A	NES (2)	VRGLGLPKLYL	[37]	ValidNES
CDKN1A	NLS	RKRRQTSMTDFYHSKR	[37, 38]	
CDKN1B	NES	LFGPVDHEELTRDL	[39]	ValidNES
CFL1	NES	VIKVFNDMKV	[40]	ValidNES
CFL1	NLS	RKSSTPEEVKKRKK	[40-42]	LocSigDB
CFLAR	NES	LLDLHIEL	[43]	ValidNES
CFLAR	NLS	RKRPLLDLHIELNGYMYDWNSRVSAKEKYYVWLQHTLRKKL	[43]	
CLIC4	NLS	KVVAKKYRN	[44, 45]	
CLK3	NLS	RSPSFGEDYYGPSRSRHRRRSRERGPY	[34]	
CPEB1	NES (1)	LSDLISSLRI	[46]	ValidNES
CPEB1	NES (2)	LCLGLQSLSL	[46]	ValidNES
CPSF6	NLS	PPTNSGMPTSDSRGPPPTDPYGRPPPY	[34]	
CRY2	NLS	PKRKLEAAEEPPGEELSKRAR	[47]	
CTNNBL1	NLS	RGTKRPRDDEEEEQKMRRK	[48]	
CTNND1	NES	ESLEEELDVLVLDDEGG	[49]	ValidNES
CUL4B	NLS	KKRK	[50]	LocSigDB
DAG1	NLS	RKKRKGK	[51]	
DBR1	NLS	KRLSDEHEPEQRKKIKRR	[52]	
DCAF8	NES	IEVEASDLSLSL	[53]	
DCAF8	NLS	PRRRVQRKR	[53]	
DEAF1	NLS	KRRKK	[54]	
DEAF1	NES	WLYLEEMVNSLLNTAQQLKTLFEQ	[54]	ValidNES
DGKZ	NLS	KASKKKKRASFKRKSSKK	[55]	
DKC1	NLS	KKEKKKSKK	[56]	LocSigDB, NLSdb

DOK1	NES	LLKAKLTDPKED	[57]	ValidNES
DOK7	NES	QLEKRLSLLS	[58]	ValidNES
DR1	NES	VIQALESLGF	[59]	ValidNES
DR1	NLS	KRRK	[59]	
DST	NLS	PVKRRRM	[60]	
DUSP16	NES	LVQALSGLHL	[61]	ValidNES
DUSP16	NLS	KKIKNQTGASGPKSKLK	[61]	
DYRK4	NLS	KKPRKCDLTPFLVLKARKKQ	[62]	
ECT2	NLS	RKRRRLKETLAQLSRETDVSPFPPRKR	[63]	
EGFR	NLS	RRRHIVRKRTLRR	[64]	LocSigDB
EIF3E	NLS	RKRRQVLKDLVKVIQQESYTYKDPITEFVECLYVNFDFDGAQKKLR	[65]	
EIF3E	NES	LTTRIAHFLDRHLV	[65]	ValidNES
ELAVL1	NLS	HHQAQRFRFSPMGVDHMSGLSGVNVPG	[34]	
ELF3	NES	LCNCALEELRL	[66]	ValidNES
ELF3	NES	LWEFIRDILI	[66]	ValidNES
ELF3	NLS	HGKRKR	[66]	
ESR1	NES	EFVCLKSIILLNS	[67]	ValidNES
EWSR1	NLS	GGRRGGRGGPGKMDKGEHRQERRDRPY	[34]	
EXO1	NLS	KRPR	[68]	LocSigDB
FGF1	NES	ILFLPLPV	[69]	ValidNES
FGF1	NLS	NYKKPKL	[70]	LocSigDB
FGF2	NLS	RSRKYTSWYVALKR	[71]	
FGF3	NLS	RLRRDAGGRGGVYEHLGGAPRRR	[72]	LocSigDB
FHL1	NLS	KRTVSRVSHPVSKARK	[73]	
FHL1	NES	LPLTLFPSANL	[74]	ValidNES
FMR1	NES	EVDQLRLERLQIDEQLR	[75]	ValidNES

FOXO3	NES	LTDMAGTMNLNDGLTENLMDDLLDNITL	[76]	ValidNES
FOXO3	NLS	RRRA	[76]	
FUS	NLS	GGDRGGFGPGKMDSRGEHRQDRRERPY	[34]	
GABPB1	NLS	EEPPAKRQCIEI	[77]	
GAPDH	NES	KKVVKQASEGPLK	[78]	ValidNES
GPR158	NLS	LKKLYAQLEIYKRKK	[79]	
GRK4	NLS	QKKRIKKRK	[80]	
GRK5	NLS	RKEKVKRE	[80]	
GRK6	NLS	RKKKIKRE	[80]	
HDAC4	NLS	KLRSRLKQKVAERRSSPLLRRK	[81]	
HDAC4	NES	VTAMASLSVGVKPA	[81, 82]	ValidNES
HDAC5	NES	VSAMALLSVGAEQAQAAAAREHSPRPAEEPMEQEPAL	[82]	ValidNES
HDAC5	NLS	EPNLKVRSRLKQKVAERRSSPLLRRKDGTVISTFKKRAVEITGAG	[82, 83]	
HEXIM1	NLS	WGQQQRQLGKKKHRRRPSKKKRHWKPY	[34]	
HEY1	NLS	ARKRRR	[84]	
HIF1A	NES	MEDIKILI	[85]	ValidNES
HNRNPA0	NLS	SYGPMKSGGGGGGGGSSWGGRSNSGPY	[34]	
HNRNPA1	NLS	FGNYNNQSSNFGPMKGGNFGGRSSGPY	[34, 86]	LocSigDB
HNRNPA2B1	NLS	NYNQQPSNYGPMKSGNFGGSRNMGGPY	[34]	
HNRNPA3	NLS	NYSGQQQSNYGPMKGGSFGGRSSGSPY	[34]	
HNRNPD	NLS	YGDYSNQQSGYGKVSRRGGHQNSYKPY	[34]	LocSigDB
HNRNPF	NLS	FKSSQEEVRSYSDPPLKFMSVQRPGPY	[34]	LocSigDB
HNRNPM	NLS	GEGERPAQNEKRKEKNIKRGGNRFEPY	[34]	LocSigDB
HSPA8	NLS	KRKHKKDISENKRAVRR	[87, 88]	LocSigDB
HSPA8	NES	LDVTPLSL	[87]	ValidNES
HSH2D	NES	LWRSLKML	[89]	ValidNES

HTT	NES	IIISLARLPL	[90]	ValidNES
IFI16	NLS	QKRKKSTKEKAGPKGSKVS	[91]	
IGFBP2	NLS	PKKLRPPP	[92]	LocSigDB
IL5	NLS	KKYIDGQKKKCGEERRRV	[93]	LocSigDB
ILKAP	NLS	KTEGKGAKRKTSEEEK	[94]	
INHBA	NLS	KKKKKEEEGEGKKK	[95]	LocSigDB
IPMK	NLS	RHRKIYTKKHH	[96, 97]	
IPMK	NES	LMEEIGFLVL	[97]	ValidNES
IRF2BP2	NLS	ARKRKPSP	[98]	LocSigDB
IRF3	NES	ILDELLGNMVL	[99]	ValidNES
ITPKB	NES	LQRELQNVQV	[100]	ValidNES
ITPKB	NLS	KRKLR	[100]	
JUN	NLS	RKRKL	[101]	LocSigDB
KANK1	NLS (1)	KRRK	[102]	
KANK1	NLS (2)	KKKDGNKDSNGAKK	[102]	
KANK1	NES	LTLLKTNLNL	[102]	ValidNES
KDM8	NLS	KRPARGSLPEQPCTKKARA	[103]	
KDM8	NES	EDLKLDL	[103]	
KEAP1	NES	LVKIFEELTL	[104]	ValidNES
KHDRBS1	NLS	DDWNGTRPSLKAPPARPVKGAYREHPY	[34]	
KIF4A	NLS	PKLRRR	[105]	
KLF6	NLS	CHFNGCRKVYTKSSHLKAHQRTHT	[106]	
KLF6	NES	MDVLPMCSIFQELQIV	[106]	ValidNES
LPIN1	NLS	KKRRKRRRK	[107]	
LZTS2	NES	LEQELQQLSLEL	[97]	ValidNES
MALT1	NES (1)	LTNLLRQL	[108]	ValidNES

MALT1	NES (2)	IPILDAL	[108]	ValidNES
MAX	NLS	PQSRKKLR	[109]	LocSigDB, NLSdb
MCM3	NLS	KKVLEKEKKRKKRSEDESETEDEEEKSQEDQEQKRKRRKTR	[110]	
MDM2	NES	SISLSFDESLALCVI	[111, 112]	ValidNES
MIER1	NES	FTDCLWTLFL	[113]	ValidNES
MIER1	NLS	RPAKRRR	[113]	
MSH5	NES	LVDKFMKLDL	[114]	ValidNES
MSH5	NLS	KKRR	[114]	
MTF1	NLS	KRKEVKR	[115]	
MTF1	NES	LCLSDLSLL	[115]	ValidNES
MTOR	NES (1)	LEWLRRLSL	[116]	ValidNES
MTOR	NES (2)	IQDGLLKMLSLVL	[116]	ValidNES
MTOR	NES (3)	ITFIFKSLGL	[116]	ValidNES
MYB	NLS	PPLLKKIKQ	[117]	LocSigDB, NLSdb
MYC	NLS	PAAKRVKLD	[118]	LocSigDB
MYO1C	NLS	GRRKAAKRKWAAQ	[119]	LocSigDB
NACC1	NLS	KRLWDSGQKEAGGGGNGSRKMAK	[120]	
NANOG	NES	MQELSNILNL	[121]	ValidNES
NANOG	NLS (1)	RMKSKRWQ	[121, 122]	
NANOG	NLS (2)	KKQKTR	[121, 122]	
NEDD4	NES	LAEELNARLTI	[123]	ValidNES
NFAT5	NLS	RKSRKR	[124]	
NFAT5	NES	MPSDFISLLSADLDLESP	[124]	ValidNES
NFE2	NLS	RRRGKNKVAAQNCRKRK	[125]	
NFE2L1	NES	LSLEECLRLL	[126]	ValidNES
NFE2L2	NES (1)	LLSIPELQCLNI	[127, 128]	ValidNES

NFE2L2	NES (2)	LKKQLSTLYL	[127, 128]	ValidNES
NFKB1	NLS	RKRQK	[129]	LocSigDB, NLSdb
NFKBIA	NES	MVKELQEIRL	[130]	ValidNES
NFKBIE	NES	VLLPFDDLKI	[131]	ValidNES
NKX2-5	NLS	RRRRKPR	[132]	
NMRAL1	NES	IELTLRL	[133]	ValidNES
NOTCH1	NLS (1)	KKFRFE	[134]	
NOTCH1	NLS (2)	RKRRR	[134]	
NPM1	NLS	KRSAPGGGSKVPQKKVK	[135]	
NPM1	NES (1)	LSLRTVSL	[135-137]	ValidNES
NPM1	NES (2)	ITPPVVLRL	[135-137]	ValidNES
NR3C1	NLS	RKCLQAGMNLEARKTKK	[21, 138]	LocSigDB, NLSdb
NUCKS1	NLS	KKIR	[139]	LocSigDB
NXF1	NLS	SSRLEEDDGDVAMSDAQDGPRVRYNPY	[34]	
PABPN1	NLS	FYSGFNSRPRGRVYRGRARATSWYSPY	[34]	
PAK1	NLS (1)	KKKPKMSDEEILEKLRSIVSVGDPKKK	[140]	
PAK1	NLS (2)	EKKKKDRFYRSILPGDKTNKKKE	[140]	
PATL1	NES	LAERLSKMVI	[141]	ValidNES
PARP1	NLS	KRKGDEVDGVDEVAKKKSKK	[142]	LocSigDB
PCNT	NES (1)	LQDALRRLLGL	[143]	ValidNES
PCNT	NES (2)	FGETLRAAVTL	[143]	ValidNES
PCNT	NES (3)	LDEFNELAI	[143]	ValidNES
PCNT	NES (4)	VIEKLQHELSL	[143]	ValidNES
PCNT	NES (5)	LELEALRLSL	[143]	ValidNES
PCNT	NLS	RRRKVEAGRTKLAHFRQRKTKGDSSHSEKKTAKRK	[143]	LocSigDB
PDGFB	NLS	RVTIRTVRVRRPPKGKHRK	[144]	LocSigDB

PDX1	NLS	RRMKWKK	[145]	LocSigDB, NLSdb
PHB	NES	ITYLPAGQSVLLQL	[146]	ValidNES
PKIA	NES	LALKLAGLDI	[147, 148]	ValidNES
PLK1	NLS	RQEEAEDPACIPIFWVSKWVDYSDKYGLGYQLCDNSVG	[149]	
PLSCR1	NLS	GKISKHWTGI	[150]	LocSigDB
PLSCR4	NLS	GSIIRKWN	[151]	LocSigDB
POU3F1	NLS	GRKRKKRT	[152]	LocSigDB
POU5F1	NLS	RKRKR	[153]	
PPP2CA	NES	LFDYLPLTAL	[154]	ValidNES
PPP2R5A	NES	LWKKLEELKL	[155]	ValidNES
PQBP1	NLS	RDRGYDKADREEGKERRHHRREELAPY	[34]	LocSigDB
PRKD2	NLS	RKRR	[156]	
PRKD2	NES	GEMLFGLVRQGL	[156]	ValidNES
PRKG1	NLS	KILKKRHI	[157]	LocSigDB
PTHLH	NLS	RYLTQETNKVETYKEQPLKTPGKKKKGK	[158]	LocSigDB, NLSdb
PTHLH	NES	LSDTSTTSLEL	[159]	ValidNES
PTK2	NES	LDLASLIL	[160]	
PTMA	NLS	TKKQKT	[161]	LocSigDB
PTPN2	NLS	RKRKR	[162]	LocSigDB
RANBP1	NES	EKVAEKLEALSVKEETKE	[163, 164]	ValidNES
RANBP3	NLS	PPVKRERTS	[118]	LocSigDB, NLSdb
RB1	NLS	KRSAEGSNPPKPLKKLR	[165-167]	LocSigDB, NLSdb
RBM15B	NLS	GLPKPWEERRKRRSLSSDRGRTTHSPY	[34]	
RBM39	NLS	RSRSKERRRSRSRSRDRRFRGRYRSPY	[34]	
RCC1	NLS	KRIAKRRSPPADAIPKSKKVK	[168]	
RELA	NLS	EEKRKR	[129]	LocSigDB, NLSdb

RELA	NES	LSEALLQLQF	[169]	ValidNES
RIPK3	NES	LEGLKELMQL	[170]	ValidNES
RIPK3	NES	MVSEWLNKLNL	[170]	ValidNES
RIPK3	NLS	NPVTGRPLVNIYNCSGVQVGDNNYLTMQQTT	[170]	
RNMT	NLS	KKRKLDPEIVPEEKDCGDAEGNSKKRKR	[171]	
RPS25	NLS	DPVNKSGGKAKKKKWSKGKVRD	[172]	
SAMHD1	NLS	KRPR	[173, 174]	
SECISBP2	NLS	KKKK	[175]	
SECISBP2	NES	QMLSKEVDACVTDLLKELVRFQD	[175]	ValidNES
SENP2	NES	LEPDLSEEVSARLRL	[176]	ValidNES
SENP2	NLS	KRRRSDSTLFSTVDTDEIPAKRPR	[176]	
SERTAD2	NES	LTDLTL	[177]	ValidNES
SH2B1	NLS	KPKLKKR	[178]	
SIRT2	NES	LRNLFSQTLSL	[179]	ValidNES
SIRT7	NLS	LQGRSRRREGLKRRQE	[180]	
SMAD1	NES	LTKMCTIRM	[181]	ValidNES
SMAD1	NLS	KKLKKKK	[181]	LocSigDB
SMAD3	NLS	KKLKKT	[182]	LocSigDB
SMAD4	NES	GIDLSGLTL	[183]	ValidNES
SMARCB1	NES	IIKLNIHVGNISLV	[184]	ValidNES
SOCS1	NLS	RRMLGAPLRQRRVR	[185]	LocSigDB
SOX14	NLS	HPDYKYRPRRKPKNLLKKDRYVFPLPY	[34]	
SOX9	NLS (1)	KRPMNAFMVWAQAARRK	[186]	LocSigDB, NLSdb
SOX9	NLS (2)	PRRRK	[186]	LocSigDB, NLSdb

SPAST	NLS	RKKK	[187, 188]	
SPAST	NLS	RGKKK	[187]	
SRF	NLS	RRGLKR	[189]	LocSigDB
SRY	NLS (1)	RPRRK	[190]	LocSigDB, NLSdb
SRY	NLS (2)	KRPMNAFIVWSRDQRRK	[190]	LocSigDB, NLSdb
STAG2	NLS	KNQKQGKGKTCKKGKK	[191]	LocSigDB
STAT1	NES	LLLKKMYL	[43]	ValidNES
STK4	NES (1)	LPSQLGTMVI	[192]	ValidNES
STK4	NES (2)	LKSWTVEDLQKRL	[192]	ValidNES
STK4	NLS	KRQPILDAIEAKKRR	[192]	
TAF15	NLS	YGGDRGGYGGKMGGRNDYRNDQRNRPY	[34]	
TFEB	NLS	IERRRR	[193]	
TFIP11	NLS	VKDKFN	[194]	
THRB	NLS	KRLAKRKLIEENREKRRR	[195]	LocSigDB

TMOD1	NES	ELCDIAAIL	[196]	ValidNES
TMOD1	NLS	RKRR	[196]	
TOB1	NES	QLEIQVALNFIIS	[197]	ValidNES
TOB1	NLS	NKLPRRRVNIFGEELERLLKKKY	[197]	
TOP1	NLS	NKKKKPKKEEEQKWKWWEEERYPEGIKW	[198]	
TOP2A	NLS	KPDPAKTKNRRKRKPSTSDDSDSNFEKIVSKAVTSKKSKGESD	[199]	
TOP2A	NES	ILRDFFELRLK	[200]	ValidNES
TOP2B	NES	ILKEFFDLRLS	[200]	ValidNES
TP53	NLS	TKRALPNNTSSSPQPKKK	[201]	LocSigDB, NLSdb
TP53	NES	MFRELNEALEL	[111, 202, 203]	ValidNES
TP73	NES	ILMKLKESLEL	[203]	ValidNES
TP73	NLS	KRAFKQSPPAVPALGAGVKKRR	[203]	
TRIM27	NES	LEELDL	[204]	ValidNES
USP21	NES	ELGAALSRLALRPEPPTLR	[205]	ValidNES

USP22	NLS	KRELELLKHNPKRRKIT	[206]	LocSigDB
VDR	NLS	RRSMKRK	[207]	LocSigDB, NLSdb
WASL	NES	LNNLDPELKNL	[208]	ValidNES
WASL	NLS	KKFRKAVTDLLGRRQRKSEK	[208]	
WBP11	NLS	KKNKK	[209]	
WBP11	NLS	KRKKL	[209]	
WBP11	NES	DDVYEAFMKEMEGLL	[209]	ValidNES
WBSCR16	NLS	PSFVVPSSGPGPRAGARPRRRIQPVPY	[34]	
WEE1	NES	PHKTFRKLRL	[54]	ValidNES
XPA	NLS	RKRQR	[210]	
YBX1	NLS	SAPEGQAQQRRPYRRRRFPPY	[34]	
ZNF131	NLS (1)	KKQRTGKKIH	[211]	
ZNF131	NLS (2)	GKNEAKKRKIA	[211]	
ZNF655	NES	LERLQEILRKFLYL	[212]	ValidNES

[a] When more than one motif of the same type are validated in one gene, their motif type is followed by a number to uniquely identify the individual motifs. The same number is used in the heatmaps (Figures 6, 7, S2 and S3).

[b] NLS and NES databases considered: for NESs, ValidNES [213]; for NLSs, NLSdb [214] and LocSigDB [215].

References

1. Chen LY, Liu D, Songyang Z: **Telomere maintenance through spatial control of telomeric proteins**. *Mol Cell Biol* 2007, **27**(16):5898-5909.

2. Maas S, Gommans WM: **Identification of a selective nuclear import signal in adenosine deaminases acting on RNA**. *Nucleic Acids Res* 2009, **37**(17):5822-5829.
3. Chen CL, Lin YP, Lai YC, Chen HC: **alpha-Adducin translocates to the nucleus upon loss of cell-cell adhesions**. *Traffic* 2011, **12**(10):1327-1340.
4. Ikuta T, Eguchi H, Tachibana T, Yoneda Y, Kawajiri K: **Nuclear localization and export signals of the human aryl hydrocarbon receptor**. *J Biol Chem* 1998, **273**(5):2895-2904.
5. Ito S, Nagaoka H, Shinkura R, Begum N, Muramatsu M, Nakata M, Honjo T: **Activation-induced cytidine deaminase shuttles between nucleus and cytoplasm like apolipoprotein B mRNA editing catalytic polypeptide 1**. *Proc Natl Acad Sci U S A* 2004, **101**(7):1975-1980.
6. Saji M, Vasko V, Kada F, Allbritton EH, Burman KD, Ringel MD: **Akt1 contains a functional leucine-rich nuclear export sequence**. *Biochem Biophys Res Commun* 2005, **332**(1):167-173.
7. Zhang A, Li CW, Tsai SC, Chen JD: **Subcellular localization of ankyrin repeats cofactor-1 regulates its corepressor activity**. *Journal of cellular biochemistry* 2007, **101**(5):1301-1315.
8. Fries B, Heukeshoven J, Hauber I, Gruttner C, Stocking C, Kehlenbach RH, Hauber J, Chemnitz J: **Analysis of nucleocytoplasmic trafficking of the HuR ligand APRIL and its influence on CD83 expression**. *J Biol Chem* 2007, **282**(7):4504-4515.
9. Liu J, Vishwanatha JK: **Regulation of nucleo-cytoplasmic shuttling of human annexin A2: a proposed mechanism**. *Molecular and cellular biochemistry* 2007, **303**(1-2):211-220.
10. Neufeld KL, Nix DA, Bogerd H, Kang Y, Beckerle MC, Cullen BR, White RL: **Adenomatous polyposis coli protein contains two nuclear export signals and shuttles between the nucleus and cytoplasm**. *Proc Natl Acad Sci U S A* 2000, **97**(22):12085-12090.
11. Lee DK, Lanca AJ, Cheng R, Nguyen T, Ji XD, Gobeil F, Jr., Chemtob S, George SR, O'Dowd BF: **Agonist-independent nuclear localization of the Apelin, angiotensin AT1, and bradykinin B2 receptors**. *J Biol Chem* 2004, **279**(9):7901-7908.
12. Jenster G, Trapman J, Brinkmann AO: **Nuclear import of the human androgen receptor**. *The Biochemical journal* 1993, **293 (Pt 3)**:761-768.
13. Eguchi H, Ikuta T, Tachibana T, Yoneda Y, Kawajiri K: **A nuclear localization signal of human aryl hydrocarbon receptor nuclear translocator/hypoxia-inducible factor 1beta is a novel bipartite type recognized by the two components of nuclear pore-targeting complex**. *J Biol Chem* 1997, **272**(28):17640-17647.
14. Kwon I, Lee J, Chang SH, Jung NC, Lee BJ, Son GH, Kim K, Lee KH: **BMAL1 shuttling controls transactivation and degradation of the CLOCK/BMAL1 heterodimer**. *Mol Cell Biol* 2006, **26**(19):7318-7330.
15. Hoeppner CZ, Cheng N, Ye RD: **Identification of a nuclear localization sequence in beta-arrestin-1 and its functional implications**. *J Biol Chem* 2012, **287**(12):8932-8943.
16. Liu H, Deng X, Shyu YJ, Li JJ, Taparowsky EJ, Hu CD: **Mutual regulation of c-Jun and ATF2 by transcriptional activation and subcellular localization**. *EMBO J* 2006, **25**(5):1058-1069.
17. Mo P, Wang H, Lu H, Boyd DD, Yan C: **MDM2 mediates ubiquitination and degradation of activating transcription factor 3**. *J Biol Chem* 2010, **285**(35):26908-26915.
18. Taylor J, Grote SK, Xia J, Vandelft M, Graczyk J, Ellerby LM, La Spada AR, Truant R: **Ataxin-7 can export from the nucleus via a conserved exportin-dependent signal**. *J Biol Chem* 2006, **281**(5):2730-2739.

19. Manchen ST, Hubberstey AV: **Human Scythe contains a functional nuclear localization sequence and remains in the nucleus during staurosporine-induced apoptosis**. *Biochem Biophys Res Commun* 2001, **287**(5):1075-1082.
20. Engelsma D, Rodriguez JA, Fish A, Giaccone G, Fornerod M: **Homodimerization antagonizes nuclear export of survivin**. *Traffic* 2007, **8**(11):1495-1502.
21. Kaneko H, Orii KO, Matsui E, Shimozawa N, Fukao T, Matsumoto T, Shimamoto A, Furuichi Y, Hayakawa S, Kasahara K *et al*: **BLM (the causative gene of Bloom syndrome) protein translocation into the nucleus by a nuclear localization signal**. *Biochem Biophys Res Commun* 1997, **240**(2):348-353.
22. Bartholomeusz G, Wu Y, Ali Seyed M, Xia W, Kwong KY, Hortobagyi G, Hung MC: **Nuclear translocation of the pro-apoptotic Bcl-2 family member Bok induces apoptosis**. *Molecular carcinogenesis* 2006, **45**(2):73-83.
23. Thompson ME, Robinson-Benion CL, Holt JT: **An amino-terminal motif functions as a second nuclear export sequence in BRCA1**. *J Biol Chem* 2005, **280**(23):21854-21857.
24. Rodriguez JA, Henderson BR: **Identification of a functional nuclear export sequence in BRCA1**. *J Biol Chem* 2000, **275**(49):38589-38596.
25. Thakur S, Zhang HB, Peng Y, Le H, Carroll B, Ward T, Yao J, Farid LM, Couch FJ, Wilson RB *et al*: **Localization of BRCA1 and a splice variant identifies the nuclear localization signal**. *Mol Cell Biol* 1997, **17**(1):444-452.
26. Han X, Saito H, Miki Y, Nakanishi A: **A CRM1-mediated nuclear export signal governs cytoplasmic localization of BRCA2 and is essential for centrosomal localization of BRCA2**. *Oncogene* 2008, **27**(21):2969-2977.
27. Yano K, Morotomi K, Saito H, Kato M, Matsuo F, Miki Y: **Nuclear localization signals of the BRCA2 protein**. *Biochem Biophys Res Commun* 2000, **270**(1):171-175.
28. Fukazawa H, Masumi A: **The conserved 12-amino acid stretch in the inter-bromodomain region of BET family proteins functions as a nuclear localization signal**. *Biological & pharmaceutical bulletin* 2012, **35**(11):2064-2068.
29. Wu J, Wang Y, Qiao X, Saiyin H, Zhao S, Qiao S, Wu Y: **Cloning and characterization of a novel human BRMS1 transcript variant in hepatocellular carcinoma cells**. *Cancer Lett* 2013, **337**(2):266-275.
30. Rivera J, Megias D, Navas C, Bravo J: **Identification of essential sequences for cellular localization in BRMS1 metastasis suppressor**. *PLoS One* 2009, **4**(7):e6433.
31. Hurst DR, Xie Y, Thomas JW, Liu J, Edmonds MD, Stewart MD, Welch DR: **The C-terminal putative nuclear localization sequence of breast cancer metastasis suppressor 1, BRMS1, is necessary for metastasis suppression**. *PLoS One* 2013, **8**(2):e55966.
32. Li FQ, Mofunanya A, Fischer V, Hall J, Takemaru K: **Nuclear-cytoplasmic shuttling of Chibby controls beta-catenin signaling**. *Molecular biology of the cell* 2010, **21**(2):311-322.
33. Toyoshima F, Moriguchi T, Wada A, Fukuda M, Nishida E: **Nuclear export of cyclin B1 and its possible role in the DNA damage-induced G2 checkpoint**. *EMBO J* 1998, **17**(10):2728-2735.
34. Suel KE, Gu H, Chook YM: **Modular organization and combinatorial energetics of proline-tyrosine nuclear localization signals**. *PLoS biology* 2008, **6**(6):e137.
35. Bembenek J, Kang J, Kurischko C, Li B, Raab JR, Belanger KD, Luca FC, Yu H: **Crm1-mediated nuclear export of Cdc14 is required for the completion of cytokinesis in budding yeast**. *Cell cycle* 2005, **4**(7):961-971.

36. Kim BJ, Kim SY, Lee H: **Identification and characterization of human cdc7 nuclear retention and export sequences in the context of chromatin binding**. *J Biol Chem* 2007, **282**(41):30029-30038.
37. Hwang CY, Kim IY, Kwon KS: **Cytoplasmic localization and ubiquitination of p21(Cip1) by reactive oxygen species**. *Biochem Biophys Res Commun* 2007, **358**(1):219-225.
38. Poon RY, Hunter T: **Expression of a novel form of p21Cip1/Waf1 in UV-irradiated and transformed cells**. *Oncogene* 1998, **16**(10):1333-1343.
39. Connor MK, Kotchetkov R, Cariou S, Resch A, Lupetti R, Beniston RG, Melchior F, Hengst L, Slingerland JM: **CRM1/Ran-mediated nuclear export of p27(Kip1) involves a nuclear export signal and links p27 export and proteolysis**. *Molecular biology of the cell* 2003, **14**(1):201-213.
40. Munsie LN, Desmond CR, Truant R: **Cofilin nuclear-cytoplasmic shuttling affects cofilin-actin rod formation during stress**. *J Cell Sci* 2012, **125**(Pt 17):3977-3988.
41. Abe H, Nagaoka R, Obinata T: **Cytoplasmic localization and nuclear transport of cofilin in cultured myotubes**. *Exp Cell Res* 1993, **206**(1):1-10.
42. Iida K, Matsumoto S, Yahara I: **The KKRKK sequence is involved in heat shock-induced nuclear translocation of the 18-kDa actin-binding protein, cofilin**. *Cell structure and function* 1992, **17**(1):39-46.
43. Katayama R, Ishioka T, Takada S, Takada R, Fujita N, Tsuruo T, Naito M: **Modulation of Wnt signaling by the nuclear localization of cellular FLIP-L**. *J Cell Sci* 2010, **123**(Pt 1):23-28.
44. Mynott AV, Harrop SJ, Brown LJ, Breit SN, Kobe B, Curmi PM: **Crystal structure of importin-alpha bound to a peptide bearing the nuclear localisation signal from chloride intracellular channel protein 4**. *FEBS J* 2011, **278**(10):1662-1675.
45. Suh KS, Mutoh M, Nagashima K, Fernandez-Salas E, Edwards LE, Hayes DD, Crutchley JM, Marin KG, Dumont RA, Levy JM *et al*: **The organellular chloride channel protein CLIC4/mtCLIC translocates to the nucleus in response to cellular stress and accelerates apoptosis**. *J Biol Chem* 2004, **279**(6):4632-4641.
46. Ernoult-Lange M, Wilczynska A, Harper M, Aigueperse C, Dautry F, Kress M, Weil D: **Nucleocytoplasmic traffic of CPEB1 and accumulation in Crm1 nucleolar bodies**. *Molecular biology of the cell* 2009, **20**(1):176-187.
47. Sakakida Y, Miyamoto Y, Nagoshi E, Akashi M, Nakamura TJ, Mamine T, Kasahara M, Minami Y, Yoneda Y, Takumi T: **Importin alpha/beta mediates nuclear transport of a mammalian circadian clock component, mCRY2, together with mPER2, through a bipartite nuclear localization signal**. *J Biol Chem* 2005, **280**(14):13272-13278.
48. Ganesh K, Adam S, Taylor B, Simpson P, Rada C, Neuberger M: **CTNNBL1 is a novel nuclear localization sequence-binding protein that recognizes RNA-splicing factors CDC5L and Prp31**. *J Biol Chem* 2011, **286**(19):17091-17102.
49. van Hengel J, Vanhoenacker P, Staes K, van Roy F: **Nuclear localization of the p120(ctn) Armadillo-like catenin is counteracted by a nuclear export signal and by E-cadherin expression**. *Proc Natl Acad Sci U S A* 1999, **96**(14):7980-7985.
50. Zou Y, Mi J, Cui J, Lu D, Zhang X, Guo C, Gao G, Liu Q, Chen B, Shao C *et al*: **Characterization of nuclear localization signal in the N terminus of CUL4B and its essential role in cyclin E degradation and cell cycle progression**. *J Biol Chem* 2009, **284**(48):33320-33332.
51. Lara-Chacon B, de Leon MB, Leocadio D, Gomez P, Fuentes-Mera L, Martinez-Vieyra I, Ortega A, Jans DA, Cisneros B: **Characterization of an Importin alpha/beta-recognized nuclear localization signal in beta-dystroglycan**. *Journal of cellular biochemistry* 2010, **110**(3):706-717.

52. Kataoka N, Dobashi I, Hagiwara M, Ohno M: **hDbr1 is a nucleocytoplasmic shuttling protein with a protein phosphatase-like motif essential for debranching activity**. *Scientific reports* 2013, **3**:1090.
53. Wu F, Wang S, Xing J, Li M, Zheng C: **Characterization of nuclear import and export signals determining the subcellular localization of WD repeat-containing protein 42A (WDR42A)**. *FEBS Lett* 2012, **586**(8):1079-1085.
54. Jensik PJ, Huggenvik JI, Collard MW: **Identification of a nuclear export signal and protein interaction domains in deformed epidermal autoregulatory factor-1 (DEAF-1)**. *J Biol Chem* 2004, **279**(31):32692-32699.
55. Topham MK, Bunting M, Zimmerman GA, McIntyre TM, Blackshear PJ, Prescott SM: **Protein kinase C regulates the nuclear localization of diacylglycerol kinase-zeta**. *Nature* 1998, **394**(6694):697-700.
56. Youssoufian H, Gharibyan V, Qatanani M: **Analysis of epitope-tagged forms of the dyskeratosis congenital protein (dyskerin): identification of a nuclear localization signal**. *Blood cells, molecules & diseases* 1999, **25**(5-6):305-309.
57. Niu Y, Roy F, Saltel F, Andrieu-Soler C, Dong W, Chantegrel AL, Accardi R, Thepot A, Foiselle N, Tommasino M *et al*: **A nuclear export signal and phosphorylation regulate Dok1 subcellular localization and functions**. *Mol Cell Biol* 2006, **26**(11):4288-4301.
58. Hamuro J, Higuchi O, Okada K, Ueno M, Iemura S, Natsume T, Spearman H, Beeson D, Yamanashi Y: **Mutations causing DOK7 congenital myasthenia ablate functional motifs in Dok-7**. *J Biol Chem* 2008, **283**(9):5518-5524.
59. Kahle J, Piaia E, Neimanis S, Meisterernst M, Doenecke D: **Regulation of nuclear import and export of negative cofactor 2**. *J Biol Chem* 2009, **284**(14):9382-9393.
60. Young KG, Pool M, Kothary R: **Bpag1 localization to actin filaments and to the nucleus is regulated by its N-terminus**. *J Cell Sci* 2003, **116**(Pt 22):4543-4555.
61. Masuda K, Shima H, Watanabe M, Kikuchi K: **MKP-7, a novel mitogen-activated protein kinase phosphatase, functions as a shuttle protein**. *J Biol Chem* 2001, **276**(42):39002-39011.
62. Papadopoulos C, Arato K, Lilienthal E, Zerweck J, Schutkowski M, Chatain N, Muller-Newen G, Becker W, de la Luna S: **Splice variants of the dual specificity tyrosine phosphorylation-regulated kinase 4 (DYRK4) differ in their subcellular localization and catalytic activity**. *J Biol Chem* 2011, **286**(7):5494-5505.
63. Saito S, Liu XF, Kamijo K, Raziuddin R, Tatsumoto T, Okamoto I, Chen X, Lee CC, Lorenzi MV, Ohara N *et al*: **Deregulation and mislocalization of the cytokinesis regulator ECT2 activate the Rho signaling pathways leading to malignant transformation**. *J Biol Chem* 2004, **279**(8):7169-7179.
64. Hsu SC, Hung MC: **Characterization of a novel tripartite nuclear localization sequence in the EGFR family**. *J Biol Chem* 2007, **282**(14):10432-10440.
65. Guo J, Sen GC: **Characterization of the interaction between the interferon-induced protein P56 and the Int6 protein encoded by a locus of insertion of the mouse mammary tumor virus**. *Journal of virology* 2000, **74**(4):1892-1899.
66. Prescott JD, Poczobutt JM, Tentler JJ, Walker DM, Gutierrez-Hartmann A: **Mapping of ESE-1 subdomains required to initiate mammary epithelial cell transformation via a cytoplasmic mechanism**. *Molecular cancer* 2011, **10**:103.
67. Lombardi M, Castoria G, Migliaccio A, Barone MV, Di Stasio R, Ciociola A, Bottero D, Yamaguchi H, Appella E, Auricchio F: **Hormone-dependent nuclear export of estradiol receptor and DNA synthesis in breast cancer cells**. *The Journal of cell biology* 2008, **182**(2):327-340.

68. Knudsen NO, Nielsen FC, Vinther L, Bertelsen R, Holten-Andersen S, Liberti SE, Hofstra R, Kooi K, Rasmussen LJ: **Nuclear localization of human DNA mismatch repair protein exonuclease 1 (hEXO1)**. *Nucleic Acids Res* 2007, **35**(8):2609-2619.
69. Nilsen T, Rosendal KR, Sorensen V, Wesche J, Olsnes S, Wiedlocha A: **A nuclear export sequence located on a beta-strand in fibroblast growth factor-1**. *J Biol Chem* 2007, **282**(36):26245-26256.
70. Zhan X, Hu X, Friedman S, Maciag T: **Analysis of endogenous and exogenous nuclear translocation of fibroblast growth factor-1 in NIH 3T3 cells**. *Biochem Biophys Res Commun* 1992, **188**(3):982-991.
71. Sheng Z, Lewis JA, Chirico WJ: **Nuclear and nucleolar localization of 18-kDa fibroblast growth factor-2 is controlled by C-terminal signals**. *J Biol Chem* 2004, **279**(38):40153-40160.
72. Kiefer P, Acland P, Pappin D, Peters G, Dickson C: **Competition between nuclear localization and secretory signals determines the subcellular fate of a single CUG-initiated form of FGF3**. *EMBO J* 1994, **13**(17):4126-4136.
73. Lee SM, Li HY, Ng EK, Or SM, Chan KK, Kotaka M, Chim SS, Tsui SK, Waye MM, Fung KP *et al*: **Characterization of a brain-specific nuclear LIM domain protein (FHL1B) which is an alternatively spliced variant of FHL1**. *Gene* 1999, **237**(1):253-263.
74. Brown S, McGrath MJ, Ooms LM, Gurung R, Maimone MM, Mitchell CA: **Characterization of two isoforms of the skeletal muscle LIM protein 1, SLIM1. Localization of SLIM1 at focal adhesions and the isoform slimmer in the nucleus of myoblasts and cytoplasm of myotubes suggests distinct roles in the cytoskeleton and in nuclear-cytoplasmic communication**. *J Biol Chem* 1999, **274**(38):27083-27091.
75. Eberhart DE, Malter HE, Feng Y, Warren ST: **The fragile X mental retardation protein is a ribonucleoprotein containing both nuclear localization and nuclear export signals**. *Hum Mol Genet* 1996, **5**(8):1083-1091.
76. Brunet A, Kanai F, Stehn J, Xu J, Sarbassova D, Frangioni JV, Dalal SN, DeCaprio JA, Greenberg ME, Yaffe MB: **14-3-3 transits to the nucleus and participates in dynamic nucleocytoplasmic transport**. *The Journal of cell biology* 2002, **156**(5):817-828.
77. Hayashi R, Takeuchi N, Ueda T: **Nuclear Respiratory Factor 2beta (NRF-2beta) recruits NRF-2alpha to the nucleus by binding to importin-alpha:beta via an unusual monopartite-type nuclear localization signal**. *J Mol Biol* 2013, **425**(18):3536-3548.
78. Brown VM, Krynetski EY, Krynetskaia NF, Grieger D, Mukatira ST, Murti KG, Slaughter CA, Park HW, Evans WE: **A novel CRM1-mediated nuclear export signal governs nuclear accumulation of glyceraldehyde-3-phosphate dehydrogenase following genotoxic stress**. *J Biol Chem* 2004, **279**(7):5984-5992.
79. Patel N, Itakura T, Gonzalez JM, Jr., Schwartz SG, Fini ME: **GPR158, an orphan member of G protein-coupled receptor Family C: glucocorticoid-stimulated expression and novel nuclear role**. *PLoS One* 2013, **8**(2):e57843.
80. Johnson LR, Robinson JD, Lester KN, Pitcher JA: **Distinct structural features of G protein-coupled receptor kinase 5 (GRK5) regulate its nuclear localization and DNA-binding ability**. *PLoS One* 2013, **8**(5):e62508.
81. Wang AH, Yang XJ: **Histone deacetylase 4 possesses intrinsic nuclear import and export signals**. *Mol Cell Biol* 2001, **21**(17):5992-6005.
82. McKinsey TA, Zhang CL, Olson EN: **Identification of a signal-responsive nuclear export sequence in class II histone deacetylases**. *Mol Cell Biol* 2001, **21**(18):6312-6321.
83. McKinsey TA, Zhang CL, Lu J, Olson EN: **Signal-dependent nuclear export of a histone deacetylase regulates muscle differentiation**. *Nature* 2000, **408**(6808):106-111.

84. Villaronga MA, Lavery DN, Bevan CL, Llanos S, Belandia B: **HEY1 Leu94Met gene polymorphism dramatically modifies its biological functions**. *Oncogene* 2010, **29**(3):411-420.
85. Mylonis I, Chachami G, Paraskeva E, Simos G: **Atypical CRM1-dependent nuclear export signal mediates regulation of hypoxia-inducible factor-1alpha by MAPK**. *J Biol Chem* 2008, **283**(41):27620-27627.
86. Iijima M, Suzuki M, Tanabe A, Nishimura A, Yamada M: **Two motifs essential for nuclear import of the hnRNP A1 nucleocytoplasmic shuttling sequence M9 core**. *FEBS Lett* 2006, **580**(5):1365-1370.
87. Tsukahara F, Maru Y: **Identification of novel nuclear export and nuclear localization-related signals in human heat shock cognate protein 70**. *J Biol Chem* 2004, **279**(10):8867-8872.
88. Lamian V, Small GM, Feldherr CM: **Evidence for the existence of a novel mechanism for the nuclear import of Hsc70**. *Exp Cell Res* 1996, **228**(1):84-91.
89. Shapiro MJ, Chen YY, Shapiro VS: **The carboxyl-terminal segment of the adaptor protein ALX directs its nuclear export during T cell activation**. *J Biol Chem* 2005, **280**(46):38242-38246.
90. Xia J, Lee DH, Taylor J, Vandelft M, Truant R: **Huntingtin contains a highly conserved nuclear export signal**. *Hum Mol Genet* 2003, **12**(12):1393-1403.
91. Briggs LJ, Johnstone RW, Elliot RM, Xiao CY, Dawson M, Trapani JA, Jans DA: **Novel properties of the protein kinase CK2-site-regulated nuclear-localization sequence of the interferon-induced nuclear factor IFI 16**. *The Biochemical journal* 2001, **353**(Pt 1):69-77.
92. Azar WJ, Zivkovic S, Werther GA, Russo VC: **IGFBP-2 nuclear translocation is mediated by a functional NLS sequence and is essential for its pro-tumorigenic actions in cancer cells**. *Oncogene* 2014, **33**(5):578-588.
93. Jans DA, Briggs LJ, Gustin SE, Jans P, Ford S, Young IG: **A functional bipartite nuclear localisation signal in the cytokine interleukin-5**. *FEBS Lett* 1997, **406**(3):315-320.
94. Zhou W, Cao H, Yang X, Cong K, Wang W, Chen T, Yin H, Wu Z, Cai X, Liu T *et al*: **Characterization of nuclear localization signal in the N terminus of integrin-linked kinase-associated phosphatase (ILKAP) and its essential role in the down-regulation of RSK2 protein signaling**. *J Biol Chem* 2013, **288**(9):6259-6271.
95. Blauer M, Husgafvel S, Syvala H, Tuohimaa P, Ylikomi T: **Identification of a nuclear localization signal in activin/inhibin betaA subunit; intranuclear betaA in rat spermatogenic cells**. *Biology of reproduction* 1999, **60**(3):588-593.
96. Nalaskowski MM, Deschermeier C, Fanick W, Mayr GW: **The human homologue of yeast ArgRIII protein is an inositol phosphate multikinase with predominantly nuclear localization**. *The Biochemical journal* 2002, **366**(Pt 2):549-556.
97. Meyer R, Nalaskowski MM, Ehm P, Schroder C, Naj X, Brehm MA, Mayr GW: **Nucleocytoplasmic shuttling of human inositol phosphate multikinase is influenced by CK2 phosphorylation**. *Biological chemistry* 2012, **393**(3):149-160.
98. Teng AC, Al-Montashiri NA, Cheng BL, Lou P, Ozmizrak P, Chen HH, Stewart AF: **Identification of a phosphorylation-dependent nuclear localization motif in interferon regulatory factor 2 binding protein 2**. *PLoS One* 2011, **6**(8):e24100.
99. Yoneyama M, Suhara W, Fukuhara Y, Fukuda M, Nishida E, Fujita T: **Direct triggering of the type I interferon system by virus infection: activation of a transcription factor complex containing IRF-3 and CBP/p300**. *EMBO J* 1998, **17**(4):1087-1095.

100. Nalaskowski MM, Fliegert R, Ernst O, Brehm MA, Fanick W, Windhorst S, Lin H, Giehler S, Hein J, Lin YN *et al*: **Human inositol 1,4,5-trisphosphate 3-kinase isoform B (IP3KB) is a nucleocytoplasmic shuttling protein specifically enriched at cortical actin filaments and at invaginations of the nuclear envelope**. *J Biol Chem* 2011, **286**(6):4500-4510.
101. Waldmann I, Walde S, Kehlenbach RH: **Nuclear import of c-Jun is mediated by multiple transport receptors**. *J Biol Chem* 2007, **282**(38):27685-27692.
102. Wang Y, Kakinuma N, Zhu Y, Kiyama R: **Nucleo-cytoplasmic shuttling of human Kank protein accompanies intracellular translocation of beta-catenin**. *J Cell Sci* 2006, **119**(Pt 19):4002-4010.
103. Huang X, Zhang L, Qi H, Shao J, Shen J: **Identification and functional implication of nuclear localization signals in the N-terminal domain of JMJD5**. *Biochimie* 2013, **95**(11):2114-2122.
104. Velichkova M, Hasson T: **Keap1 regulates the oxidation-sensitive shuttling of Nrf2 into and out of the nucleus via a Crm1-dependent nuclear export mechanism**. *Mol Cell Biol* 2005, **25**(11):4501-4513.
105. Lee YM, Lee S, Lee E, Shin H, Hahn H, Choi W, Kim W: **Human kinesin superfamily member 4 is dominantly localized in the nuclear matrix and is associated with chromosomes during mitosis**. *The Biochemical journal* 2001, **360**(Pt 3):549-556.
106. Rodriguez E, Aburjania N, Priedigkeit NM, DiFeo A, Martignetti JA: **Nucleo-cytoplasmic localization domains regulate Kruppel-like factor 6 (KLF6) protein stability and tumor suppressor function**. *PLoS One* 2010, **5**(9).
107. Peterfy M, Harris TE, Fujita N, Reue K: **Insulin-stimulated interaction with 14-3-3 promotes cytoplasmic localization of lipin-1 in adipocytes**. *J Biol Chem* 2010, **285**(6):3857-3864.
108. Nakagawa M, Hosokawa Y, Yonezumi M, Izumiyama K, Suzuki R, Tsuzuki S, Asaka M, Seto M: **MALT1 contains nuclear export signals and regulates cytoplasmic localization of BCL10**. *Blood* 2005, **106**(13):4210-4216.
109. Kato GJ, Lee WM, Chen LL, Dang CV: **Max: functional domains and interaction with c-Myc**. *Genes Dev* 1992, **6**(1):81-92.
110. Takei Y, Tsujimoto G: **Identification of a novel MCM3-associated protein that facilitates MCM3 nuclear localization**. *J Biol Chem* 1998, **273**(35):22177-22180.
111. Zhang Y, Xiong Y: **A p53 amino-terminal nuclear export signal inhibited by DNA damage-induced phosphorylation**. *Science* 2001, **292**(5523):1910-1915.
112. Roth J, Dobbelstein M, Freedman DA, Shenk T, Levine AJ: **Nucleo-cytoplasmic shuttling of the hdm2 oncoprotein regulates the levels of the p53 protein via a pathway used by the human immunodeficiency virus rev protein**. *EMBO J* 1998, **17**(2):554-564.
113. Clements JA, Mercer FC, Paterno GD, Gillespie LL: **Differential splicing alters subcellular localization of the alpha but not beta isoform of the MIER1 transcriptional regulator in breast cancer cells**. *PLoS One* 2012, **7**(2):e32499.
114. Lahaye F, Lespinasse F, Staccini P, Palin L, Paquis-Flucklinger V, Santucci-Darmanin S: **hMSH5 is a nucleocytoplasmic shuttling protein whose stability depends on its subcellular localization**. *Nucleic Acids Res* 2010, **38**(11):3655-3671.
115. Saydam N, Georgiev O, Nakano MY, Greber UF, Schaffner W: **Nucleo-cytoplasmic trafficking of metal-regulatory transcription factor 1 is regulated by diverse stress signals**. *J Biol Chem* 2001, **276**(27):25487-25495.

116. Bachmann RA, Kim JH, Wu AL, Park IH, Chen J: **A nuclear transport signal in mammalian target of rapamycin is critical for its cytoplasmic signaling to S6 kinase 1**. *J Biol Chem* 2006, **281**(11):7357-7363.
117. Dang CV, Lee WM: **Nuclear and nucleolar targeting sequences of c-erb-A, c-myb, N-myc, p53, HSP70, and HIV tat proteins**. *J Biol Chem* 1989, **264**(30):18019-18023.
118. Welch K, Franke J, Kohler M, Macara IG: **RanBP3 contains an unusual nuclear localization signal that is imported preferentially by importin-alpha3**. *Mol Cell Biol* 1999, **19**(12):8400-8411.
119. Dzijak R, Yildirim S, Kahle M, Novak P, Hnilicova J, Venit T, Hozak P: **Specific nuclear localizing sequence directs two myosin isoforms to the cell nucleus in calmodulin-sensitive manner**. *PLoS One* 2012, **7**(1):e30529.
120. Okazaki K, Nakayama N, Nariai Y, Nakayama K, Miyazaki K, Maruyama R, Kato H, Kosugi S, Urano T, Sakashita G: **Nuclear localization signal in a cancer-related transcriptional regulator protein NAC1**. *Carcinogenesis* 2012, **33**(10):1854-1862.
121. Park SW, Do HJ, Huh SH, Sung B, Uhm SJ, Song H, Kim NH, Kim JH: **Identification of a putative nuclear export signal motif in human NANOG homeobox domain**. *Biochem Biophys Res Commun* 2012, **421**(3):484-489.
122. Do HJ, Lim HY, Kim JH, Song H, Chung HM, Kim JH: **An intact homeobox domain is required for complete nuclear localization of human Nanog**. *Biochem Biophys Res Commun* 2007, **353**(3):770-775.
123. Hamilton MH, Tcherepanova I, Huibregtse JM, McDonnell DP: **Nuclear import/export of hRPF1/Nedd4 regulates the ubiquitin-dependent degradation of its nuclear substrates**. *J Biol Chem* 2001, **276**(28):26324-26331.
124. Tong EH, Guo JJ, Huang AL, Liu H, Hu CD, Chung SS, Ko BC: **Regulation of nucleocytoplasmic trafficking of transcription factor OREBP/TonEBP/NFAT5**. *J Biol Chem* 2006, **281**(33):23870-23879.
125. Perdomo J, Fock EL, Kaur G, Yan F, Khachigian LM, Jans DA, Chong BH: **A monopartite sequence is essential for p45 NF-E2 nuclear translocation, transcriptional activity and platelet production**. *Journal of thrombosis and haemostasis : JTH* 2010, **8**(11):2542-2553.
126. Husberg C, Murphy P, Bjorgo E, Kalland KH, Kolsto AB: **Cellular localisation and nuclear export of the human bZIP transcription factor TCF11**. *Biochim Biophys Acta* 2003, **1640**(2-3):143-151.
127. Li W, Jain MR, Chen C, Yue X, Hebbar V, Zhou R, Kong AN: **Nrf2 Possesses a redox-insensitive nuclear export signal overlapping with the leucine zipper motif**. *J Biol Chem* 2005, **280**(31):28430-28438.
128. Li W, Yu SW, Kong AN: **Nrf2 possesses a redox-sensitive nuclear exporting signal in the Neh5 transactivation domain**. *J Biol Chem* 2006, **281**(37):27251-27263.
129. Beg AA, Ruben SM, Scheinman RI, Haskill S, Rosen CA, Baldwin AS, Jr.: **I kappa B interacts with the nuclear localization sequences of the subunits of NF-kappa B: a mechanism for cytoplasmic retention**. *Genes Dev* 1992, **6**(10):1899-1913.
130. Johnson C, Van Antwerp D, Hope TJ: **An N-terminal nuclear export signal is required for the nucleocytoplasmic shuttling of IkappaBalpha**. *EMBO J* 1999, **18**(23):6682-6693.
131. Lee SH, Hannink M: **Characterization of the nuclear import and export functions of Ikappa B(epsilon)**. *J Biol Chem* 2002, **277**(26):23358-23366.
132. Kasahara H, Izumo S: **Identification of the in vivo casein kinase II phosphorylation site within the homeodomain of the cardiac tisue-specifying homeobox gene product Csx/Nkx2.5**. *Mol Cell Biol* 1999, **19**(1):526-536.

133. Zhang M, Hu B, Li T, Peng Y, Guan J, Lai S, Zheng X: **A CRM1-dependent nuclear export signal controls nucleocytoplasmic translocation of HSCARG, which regulates NF-kappaB activity**. *Traffic* 2012, **13**(6):790-799.
134. Aster JC, Robertson ES, Hasserjian RP, Turner JR, Kieff E, Sklar J: **Oncogenic forms of NOTCH1 lacking either the primary binding site for RBP-Jkappa or nuclear localization sequences retain the ability to associate with RBP-Jkappa and activate transcription**. *J Biol Chem* 1997, **272**(17):11336-11343.
135. Wang W, Budhu A, Forgues M, Wang XW: **Temporal and spatial control of nucleophosmin by the Ran-Crm1 complex in centrosome duplication**. *Nature cell biology* 2005, **7**(8):823-830.
136. Falini B, Bolli N, Shan J, Martelli MP, Liso A, Pucciarini A, Bigerna B, Pasqualucci L, Mannucci R, Rosati R *et al*: **Both carboxy-terminus NES motif and mutated tryptophan(s) are crucial for aberrant nuclear export of nucleophosmin leukemic mutants in NPMc+ AML**. *Blood* 2006, **107**(11):4514-4523.
137. Mariano AR, Colombo E, Luzi L, Martinelli P, Volorio S, Bernard L, Meani N, Bergomas R, Alcalay M, Pelicci PG: **Cytoplasmic localization of NPM in myeloid leukemias is dictated by gain-of-function mutations that create a functional nuclear export signal**. *Oncogene* 2006, **25**(31):4376-4380.
138. Picard D, Yamamoto KR: **Two signals mediate hormone-dependent nuclear localization of the glucocorticoid receptor**. *EMBO J* 1987, **6**(11):3333-3340.
139. Grundt K, Haga IV, Huitfeldt HS, Ostvold AC: **Identification and characterization of two putative nuclear localization signals (NLS) in the DNA-binding protein NUCKS**. *Biochim Biophys Acta* 2007, **1773**(9):1398-1406.
140. Singh RR, Song C, Yang Z, Kumar R: **Nuclear localization and chromatin targets of p21-activated kinase 1**. *J Biol Chem* 2005, **280**(18):18130-18137.
141. Marnef A, Weil D, Standart N: **RNA-related nuclear functions of human Pat1b, the P-body mRNA decay factor**. *Molecular biology of the cell* 2012, **23**(1):213-224.
142. Schreiber V, Molinete M, Boeuf H, de Murcia G, Menissier-de Murcia J: **The human poly(ADP-ribose) polymerase nuclear localization signal is a bipartite element functionally separate from DNA binding and catalytic activity**. *EMBO J* 1992, **11**(9):3263-3269.
143. Liu Q, Yu J, Zhuo X, Jiang Q, Zhang C: **Pericentrin contains five NESs and an NLS essential for its nucleocytoplasmic trafficking during the cell cycle**. *Cell research* 2010, **20**(8):948-962.
144. Lee BA, Maher DW, Hannink M, Donoghue DJ: **Identification of a signal for nuclear targeting in platelet-derived-growth-factor-related molecules**. *Mol Cell Biol* 1987, **7**(10):3527-3537.
145. Moede T, Leibiger B, Pour HG, Berggren P, Leibiger IB: **Identification of a nuclear localization signal, RRMKWKK, in the homeodomain transcription factor PDX-1**. *FEBS Lett* 1999, **461**(3):229-234.
146. Rastogi S, Joshi B, Fusaro G, Chellappan S: **Camptothecin induces nuclear export of prohibitin preferentially in transformed cells through a CRM-1-dependent mechanism**. *J Biol Chem* 2006, **281**(5):2951-2959.
147. Henderson BR, Eleftheriou A: **A comparison of the activity, sequence specificity, and CRM1-dependence of different nuclear export signals**. *Exp Cell Res* 2000, **256**(1):213-224.
148. Wen W, Meinkoth JL, Tsien RY, Taylor SS: **Identification of a signal for rapid export of proteins from the nucleus**. *Cell* 1995, **82**(3):463-473.

149. Lee MS, Huang YH, Huang SP, Lin RI, Wu SF, Li C: **Identification of a nuclear localization signal in the polo box domain of Plk1**. *Biochim Biophys Acta* 2009, **1793**(10):1571-1578.
150. Chen MH, Ben-Efraim I, Mitrousis G, Walker-Kopp N, Sims PJ, Cingolani G: **Phospholipid scramblase 1 contains a nonclassical nuclear localization signal with unique binding site in importin alpha**. *J Biol Chem* 2005, **280**(11):10599-10606.
151. Lott K, Bhardwaj A, Sims PJ, Cingolani G: **A minimal nuclear localization signal (NLS) in human phospholipid scramblase 4 that binds only the minor NLS-binding site of importin alpha1**. *J Biol Chem* 2011, **286**(32):28160-28169.
152. Sock E, Enderich J, Rosenfeld MG, Wegner M: **Identification of the nuclear localization signal of the POU domain protein Tst-1/Oct6**. *J Biol Chem* 1996, **271**(29):17512-17518.
153. Pan G, Qin B, Liu N, Scholer HR, Pei D: **Identification of a nuclear localization signal in OCT4 and generation of a dominant negative mutant by its ablation**. *J Biol Chem* 2004, **279**(35):37013-37020.
154. Tsuchiya A, Tashiro E, Yoshida M, Imoto M: **Involvement of protein phosphatase 2A nuclear accumulation and subsequent inactivation of activator protein-1 in leptomycin B-inhibited cyclin D1 expression**. *Oncogene* 2007, **26**(11):1522-1532.
155. Flegg CP, Sharma M, Medina-Palazon C, Jamieson C, Galea M, Brocardo MG, Mills K, Henderson BR: **Nuclear export and centrosome targeting of the protein phosphatase 2A subunit B56alpha: role of B56alpha in nuclear export of the catalytic subunit**. *J Biol Chem* 2010, **285**(24):18144-18154.
156. Auer A, von Blume J, Sturany S, von Wichert G, Van Lint J, Vandenheede J, Adler G, Seufferlein T: **Role of the regulatory domain of protein kinase D2 in phorbol ester binding, catalytic activity, and nucleocytoplasmic shuttling**. *Molecular biology of the cell* 2005, **16**(9):4375-4385.
157. Gudi T, Lohmann SM, Pilz RB: **Regulation of gene expression by cyclic GMP-dependent protein kinase requires nuclear translocation of the kinase: identification of a nuclear localization signal**. *Mol Cell Biol* 1997, **17**(9):5244-5254.
158. Klopocki E, Hennig BP, Dathe K, Koll R, de Ravel T, Baten E, Blom E, Gillerot Y, Weigel JF, Kruger G *et al*: **Deletion and point mutations of PTHLH cause brachydactyly type E**. *Am J Hum Genet* 2010, **86**(3):434-439.
159. Pache JC, Burton DW, Deftos LJ, Hastings RH: **A carboxyl leucine-rich region of parathyroid hormone-related protein is critical for nuclear export**. *Endocrinology* 2006, **147**(2):990-998.
160. Ossovskaya V, Lim ST, Ota N, Schlaepfer DD, Ilic D: **FAK nuclear export signal sequences**. *FEBS Lett* 2008, **582**(16):2402-2406.
161. Manrow RE, Sburlati AR, Hanover JA, Berger SL: **Nuclear targeting of prothymosin alpha**. *J Biol Chem* 1991, **266**(6):3916-3924.
162. Tiganis T, Flint AJ, Adam SA, Tonks NK: **Association of the T-cell protein tyrosine phosphatase with nuclear import factor p97**. *J Biol Chem* 1997, **272**(34):21548-21557.
163. Zolotukhin AS, Felber BK: **Mutations in the nuclear export signal of human ran-binding protein RanBP1 block the Rev-mediated posttranscriptional regulation of human immunodeficiency virus type 1**. *J Biol Chem* 1997, **272**(17):11356-11360.
164. Plafker K, Macara IG: **Facilitated nucleocytoplasmic shuttling of the Ran binding protein RanBP1**. *Mol Cell Biol* 2000, **20**(10):3510-3521.
165. Efthymiadis A, Shao H, Hubner S, Jans DA: **Kinetic characterization of the human retinoblastoma protein bipartite nuclear localization sequence (NLS) in vivo and in vitro. A comparison with the SV40 large T-antigen NLS**. *J Biol Chem* 1997, **272**(35):22134-22139.

166. Hu W, Kemp BE, Jans DA: **Kinetic properties of nuclear transport conferred by the retinoblastoma (Rb) NLS**. *Journal of cellular biochemistry* 2005, **95**(4):782-793.
167. Zacksenhaus E, Bremner R, Phillips RA, Gallie BL: **A bipartite nuclear localization signal in the retinoblastoma gene product and its importance for biological activity**. *Mol Cell Biol* 1993, **13**(8):4588-4599.
168. Friedrich B, Quensel C, Sommer T, Hartmann E, Kohler M: **Nuclear localization signal and protein context both mediate importin alpha specificity of nuclear import substrates**. *Mol Cell Biol* 2006, **26**(23):8697-8709.
169. Harhaj EW, Sun SC: **Regulation of RelA subcellular localization by a putative nuclear export signal and p50**. *Mol Cell Biol* 1999, **19**(10):7088-7095.
170. Yang Y, Ma J, Chen Y, Wu M: **Nucleocytoplasmic shuttling of receptor-interacting protein 3 (RIP3): identification of novel nuclear export and import signals in RIP3**. *J Biol Chem* 2004, **279**(37):38820-38829.
171. Shafer B, Chu C, Shatkin AJ: **Human mRNA cap methyltransferase: alternative nuclear localization signal motifs ensure nuclear localization required for viability**. *Mol Cell Biol* 2005, **25**(7):2644-2649.
172. Kubota S, Copeland TD, Pomerantz RJ: **Nuclear and nucleolar targeting of human ribosomal protein S25: common features shared with HIV-1 regulatory proteins**. *Oncogene* 1999, **18**(7):1503-1514.
173. Guo H, Wei W, Wei Z, Liu X, Evans SL, Yang W, Wang H, Guo Y, Zhao K, Zhou JY *et al*: **Identification of critical regions in human SAMHD1 required for nuclear localization and Vpx-mediated degradation**. *PLoS One* 2013, **8**(7):e66201.
174. Brandariz-Nunez A, Valle-Casuso JC, White TE, Laguette N, Benkirane M, Brojatsch J, Diaz-Griffero F: **Role of SAMHD1 nuclear localization in restriction of HIV-1 and SIVmac**. *Retrovirology* 2012, **9**:49.
175. Papp LV, Lu J, Striebel F, Kennedy D, Holmgren A, Khanna KK: **The redox state of SECIS binding protein 2 controls its localization and selenocysteine incorporation function**. *Mol Cell Biol* 2006, **26**(13):4895-4910.
176. Itahana Y, Yeh ET, Zhang Y: **Nucleocytoplasmic shuttling modulates activity and ubiquitination-dependent turnover of SUMO-specific protease 2**. *Mol Cell Biol* 2006, **26**(12):4675-4689.
177. Cheong JK, Gunaratnam L, Hsu SI: **CRM1-mediated nuclear export is required for 26 S proteasome-dependent degradation of the TRIP-Br2 proto-oncoprotein**. *J Biol Chem* 2008, **283**(17):11661-11676.
178. Maures TJ, Su HW, Argetsinger LS, Grinstein S, Carter-Su C: **Phosphorylation controls a dual-function polybasic nuclear localization sequence in the adapter protein SH2B1beta to regulate its cellular function and distribution**. *J Cell Sci* 2011, **124**(Pt 9):1542-1552.
179. North BJ, Verdin E: **Interphase nucleo-cytoplasmic shuttling and localization of SIRT2 during mitosis**. *PLoS One* 2007, **2**(8):e784.
180. Kiran S, Chatterjee N, Singh S, Kaul SC, Wadhwa R, Ramakrishna G: **Intracellular distribution of human SIRT7 and mapping of the nuclear/nucleolar localization signal**. *FEBS J* 2013, **280**(14):3451-3466.
181. Xiao Z, Watson N, Rodriguez C, Lodish HF: **Nucleocytoplasmic shuttling of Smad1 conferred by its nuclear localization and nuclear export signals**. *J Biol Chem* 2001, **276**(42):39404-39410.
182. Xiao Z, Liu X, Henis YI, Lodish HF: **A distinct nuclear localization signal in the N terminus of Smad 3 determines its ligand-induced nuclear translocation**. *Proc Natl Acad Sci U S A* 2000, **97**(14):7853-7858.

183. Watanabe M, Masuyama N, Fukuda M, Nishida E: **Regulation of intracellular dynamics of Smad4 by its leucine-rich nuclear export signal**. *EMBO reports* 2000, **1**(2):176-182.
184. Craig E, Zhang ZK, Davies KP, Kalpana GV: **A masked NES in INI1/hSNF5 mediates hCRM1-dependent nuclear export: implications for tumorigenesis**. *EMBO J* 2002, **21**(1-2):31-42.
185. Koelsche C, Strebovsky J, Baetz A, Dalpke AH: **Structural and functional analysis of a nuclear localization signal in SOCS1**. *Molecular immunology* 2009, **46**(13):2474-2480.
186. Sudbeck P, Scherer G: **Two independent nuclear localization signals are present in the DNA-binding high-mobility group domains of SRY and SOX9**. *J Biol Chem* 1997, **272**(44):27848-27852.
187. Magariello A, Tortorella C, Patitucci A, Tortelli R, Liguori M, Mazzei R, Conforti FL, Citrigno L, Ungaro C, Simone IL *et al*: **First mutation in the nuclear localization signal sequence of spastin protein identified in a patient with hereditary spastic paraplegia**. *European journal of neurology : the official journal of the European Federation of Neurological Societies* 2013, **20**(1):e22-23.
188. Beetz C, Brodhun M, Moutzouris K, Kiehntopf M, Berndt A, Lehnert D, Deufel T, Bastmeyer M, Schickel J: **Identification of nuclear localisation sequences in spastin (SPG4) using a novel Tetra-GFP reporter system**. *Biochem Biophys Res Commun* 2004, **318**(4):1079-1084.
189. Rech J, Barlat I, Veyrune JL, Vie A, Blanchard JM: **Nuclear import of serum response factor (SRF) requires a short amino-terminal nuclear localization sequence and is independent of the casein kinase II phosphorylation site**. *J Cell Sci* 1994, **107 (Pt 11)**:3029-3036.
190. Poulat F, Girard F, Chevron MP, Goze C, Rebillard X, Calas B, Lamb N, Berta P: **Nuclear localization of the testis determining gene product SRY**. *The Journal of cell biology* 1995, **128**(5):737-748.
191. Tarnowski LJ, Kowalec P, Milewski M, Jurek M, Plochocka D, Fronk J, Kurlandzka A: **Nuclear import and export signals of human cohesins SA1/STAG1 and SA2/STAG2 expressed in Saccharomyces cerevisiae**. *PLoS One* 2012, **7**(6):e38740.
192. Lee KK, Yonehara S: **Phosphorylation and dimerization regulate nucleocytoplasmic shuttling of mammalian STE20-like kinase (MST)**. *J Biol Chem* 2002, **277**(14):12351-12358.
193. Roczniak-Ferguson A, Petit CS, Froehlich F, Qian S, Ky J, Angarola B, Walther TC, Ferguson SM: **The transcription factor TFEB links mTORC1 signaling to transcriptional control of lysosome homeostasis**. *Science signaling* 2012, **5**(228):ra42.
194. Tannukit S, Crabb TL, Hertel KJ, Wen X, Jans DA, Paine ML: **Identification of a novel nuclear localization signal and speckle-targeting sequence of tuftelin-interacting protein 11, a splicing factor involved in spliceosome disassembly**. *Biochem Biophys Res Commun* 2009, **390**(3):1044-1050.
195. Mavinakere MS, Powers JM, Subramanian KS, Roggero VR, Allison LA: **Multiple novel signals mediate thyroid hormone receptor nuclear import and export**. *J Biol Chem* 2012, **287**(37):31280-31297.
196. Kong KY, Kedes L: **Cytoplasmic nuclear transfer of the actin-capping protein tropomodulin**. *J Biol Chem* 2004, **279**(29):30856-30864.
197. Maekawa M, Yamamoto T, Nishida E: **Regulation of subcellular localization of the antiproliferative protein Tob by its nuclear export signal and bipartite nuclear localization signal sequences**. *Exp Cell Res* 2004, **295**(1):59-65.
198. Mo YY, Wang C, Beck WT: **A novel nuclear localization signal in human DNA topoisomerase I**. *J Biol Chem* 2000, **275**(52):41107-41113.
199. Mirski SE, Gerlach JH, Cummings HJ, Zirngibl R, Greer PA, Cole SP: **Bipartite nuclear localization signals in the C terminus of human topoisomerase II alpha**. *Exp Cell Res* 1997, **237**(2):452-455.

200. Mirski SE, Bielawski JC, Cole SP: **Identification of functional nuclear export sequences in human topoisomerase IIalpha and beta**. *Biochem Biophys Res Commun* 2003, **306**(4):905-911.
201. Liang SH, Clarke MF: **A bipartite nuclear localization signal is required for p53 nuclear import regulated by a carboxyl-terminal domain**. *J Biol Chem* 1999, **274**(46):32699-32703.
202. Stommel JM, Marchenko ND, Jimenez GS, Moll UM, Hope TJ, Wahl GM: **A leucine-rich nuclear export signal in the p53 tetramerization domain: regulation of subcellular localization and p53 activity by NES masking**. *EMBO J* 1999, **18**(6):1660-1672.
203. Inoue T, Stuart J, Leno R, Maki CG: **Nuclear import and export signals in control of the p53-related protein p73**. *J Biol Chem* 2002, **277**(17):15053-15060.
204. Harbers M, Nomura T, Ohno S, Ishii S: **Intracellular localization of the Ret finger protein depends on a functional nuclear export signal and protein kinase C activation**. *J Biol Chem* 2001, **276**(51):48596-48607.
205. Garcia-Santisteban I, Banuelos S, Rodriguez JA: **A global survey of CRM1-dependent nuclear export sequences in the human deubiquitinase family**. *The Biochemical journal* 2012, **441**(1):209-217.
206. Xiong J, Wang Y, Gong Z, Liu J, Li W: **Identification of a functional nuclear localization signal within the human USP22 protein**. *Biochem Biophys Res Commun* 2014, **449**(1):14-18.
207. Hsieh JC, Shimizu Y, Minoshima S, Shimizu N, Haussler CA, Jurutka PW, Haussler MR: **Novel nuclear localization signal between the two DNA-binding zinc fingers in the human vitamin D receptor**. *Journal of cellular biochemistry* 1998, **70**(1):94-109.
208. Suetsugu S, Takenawa T: **Translocation of N-WASP by nuclear localization and export signals into the nucleus modulates expression of HSP90**. *J Biol Chem* 2003, **278**(43):42515-42523.
209. Llorian M, Beullens M, Lesage B, Nicolaescu E, Beke L, Landuyt W, Ortiz JM, Bollen M: **Nucleocytoplasmic shuttling of the splicing factor SIPP1**. *J Biol Chem* 2005, **280**(46):38862-38869.
210. Li Z, Musich PR, Cartwright BM, Wang H, Zou Y: **UV-induced nuclear import of XPA is mediated by importin-alpha4 in an ATR-dependent manner**. *PLoS One* 2013, **8**(7):e68297.
211. Donaldson NS, Daniel Y, Kelly KF, Graham M, Daniel JM: **Nuclear trafficking of the POZ-ZF protein Znf131**. *Biochim Biophys Acta* 2007, **1773**(4):546-555.
212. Houlard M, Romero-Portillo F, Germani A, Depaux A, Regnier-Ricard F, Gisselbrecht S, Varin-Blank N: **Characterization of VIK-1: a new Vav-interacting Kruppel-like protein**. *Oncogene* 2005, **24**(1):28-38.
213. Fu SC, Huang HC, Horton P, Juan HF: **ValidNESs: a database of validated leucine-rich nuclear export signals**. *Nucleic Acids Res* 2013, **41**(Database issue):D338-343.
214. Nair R, Carter P, Rost B: **NLSdb: database of nuclear localization signals**. *Nucleic Acids Res* 2003, **31**(1):397-399.
215. Negi S, Pandey S, Srinivasan SM, Mohammed A, Guda C: **LocSigDB: a database of protein localization signals**. *Database : the journal of biological databases and curation* 2015, **2015**.
