# Supplementary material for: Extent of pre-translational regulation for the control of nucleocytoplasmic protein localization
Source: BMC Genomics. 2016 Jun 24;17:472. doi: 10.1186/s12864-016-2854-4 (PMC4919871; doi:10.1186/s12864-016-2854-4)
Supplement: Additional file 4: — Figure S1. Characteristics of NLS subtypes. NLSs were classified according to their type as described in the Methods. The number of NLSs of different subtype with the indicated characteristics are shown. Figure S2. Heatmap representing the level of motif inclusion for alternative NLSs. The GSE45419 RNA-seq datasets consisting of benign breast lesions, ER positive, triple negative and HER2 positive primary breast tumors (32 samples in total) were analyzed to quantify the NLS MII in each tissue considered. MII values are represented using the color scheme depicted in the legend. Genes which were detected below a threshold of 1 TPM were not further considered and are represented by grey cells in the heatmap. Only motifs present in at least 17 of the 32 samples are shown. The sample type is indicated using a color-coded bar at the top of the heatmap. While some NLSs display generally uniform MII values across the samples regardless of type, a subset show specificity for a certain sample type. In particular, the benign breast lesions cluster together and display different MII values for some NLSs, suggesting a differential regulation of the presence of the NLS in these samples. Figure S3. Heatmap representing the level of motif inclusion for alternative NESs. As for figure S2, the GSE45419 RNA-seq datasets were analyzed to quantify the NES MII in each tissue considered. The heatmap is as described for figure S2. Motifs of same type present in the same gene and displaying the exact same MII profile across all tissues were collapsed into one entry (for example, CDC7 has two annotated NESs with the same MII profile across all tissues. These motifs were collapsed into one entry labelled CDC7(1;2)). (PDF 419 kb) [file 12864_2016_2854_MOESM4_ESM.pdf]

Supplementary figures' legends

Figure S1: Characteristics of NLS subtypes. NLSs were classified according to their type as described in the Methods. The number of NLSs of different subtype with the indicated characteristics are shown.

Figure S2: Heatmap representing the level of motif inclusion for alternative NLSs. The GSE45419 RNA-seq datasets consisting of benign breast lesions, ER positive, triple negative and HER2 positive primary breast tumors (32 samples in total) were analyzed to quantify the NLS MII in each tissue considered. MII values are represented using the color scheme depicted in the legend. Genes which were detected below a threshold of 1 TPM were not further considered and are represented by grey cells in the heatmap. Only motifs present in at least 17 of the 32 samples are shown. The sample type is indicated using a color-coded bar at the top of the heatmap. While some NLSs display generally uniform MII values across the samples regardless of type, a subset show specificity for a certain sample type. In particular, the benign breast lesions cluster together and display different MII values for some NLSs, suggesting a differential regulation of the presence of the NLS in these samples.

Figure S3: Heatmap representing the level of motif inclusion for alternative NESs. As for figure S2, the GSE45419 RNA-seq datasets were analyzed to quantify the NES MII in each tissue considered. The heatmap is as described for figure S2. Motifs of same type present in the same gene and displaying the exact same MII profile across all tissues were collapsed into one entry (for example, CDC7 has two annotated NESs with the same MII profile across all tissues. These motifs were collapsed into one entry labelled CDC7(1;2)).

Additional Table 1: Regulation analysis of subclasses of NLSs

	Monopartite	Bipartite	PY-NLS	Non-classified
**Total**	**45**	**30**	**24**	**76**
Constitutive motifs	16	12	8	32
Alternative motifs	29	18	16	44
Encoded in 1 exon	39	23	15	51
Encoded in 2 exons	6	7	9	22
Encoded in 3 exons	0	0	0	3
Regulated by splicing	6	3	6	12
Regulated by alternative 5’	21	8	6	24
Regulated by alternative 3’	15	12	7	32
Regulated by frameshift	1	0	1	0

Figure S1

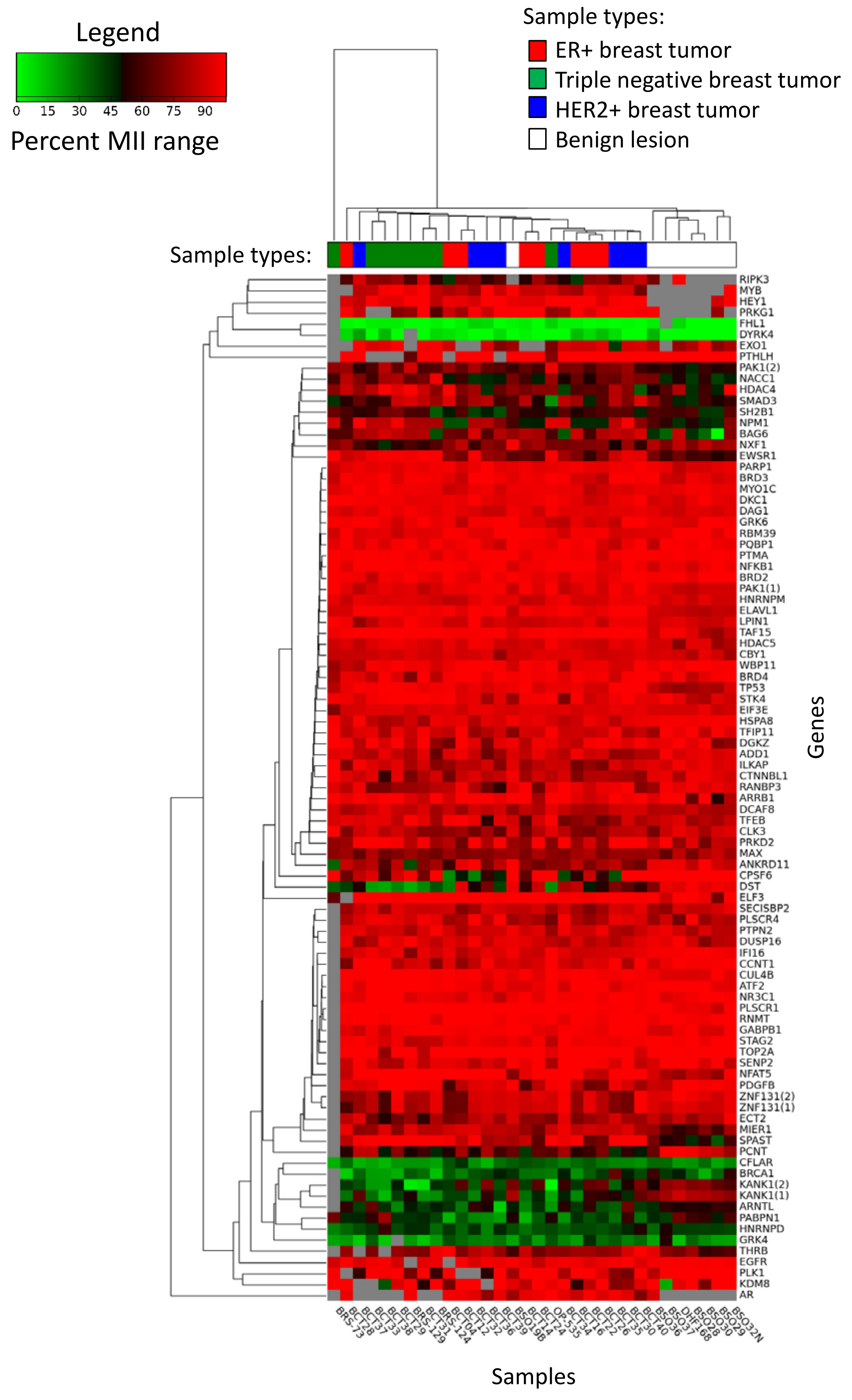
Legend
0 15 30 45 60 75 90
Percent MII range
Sample types:
ER+ breast tumor
Triple negative breast tumor
HER2+ breast tumor
Benign lesion
Sample types:
RIPK3
MYB
HEY1
PRKG1
FHL1
DYRK4
EXO1
PTHLH
PAK1(2)
NACC1
HDAC4
SMAD3
SH2B1
NPM1
BAG6
NXF1
EWSR1
PARP1
BRD3
MYO1C
DKC1
DAG1
GRK6
RBM39
PQBP1
PTMA
NFKB1
BRD2
PAK1(1)
HNRNPM
ELAVL1
LPIN1
TAF15
HDAC5
CBY1
WBP11
BRD4
TP53
STK4
EIF3E
HSPA8
TFIP11
DGKZ
ADD1
ILKAP
CTNNBL1
RANBP3
ARRB1
DCAF8
TFEB
CLK3
PRKD2
MAX
ANKRD11
CPSF6
DST
ELF3
SECISBP2
PLSCR4
PTPN2
DUSP16
IFI16
CCNT1
CUL4B
ATF2
NR3C1
PLSCR1
RNMT
GABPB1
STAG2
TOP2A
SENP2
NFAT5
PDGFB
ZNF131(2)
ZNF131(1)
ECT2
MIER1
SPAST
PCNT
CFLAR
BRCA1
KANK1(2)
KANK1(1)
ARNTL
PABPN1
HNRNPD
GRK4
THRB
EGFR
PLK1
KDM8
AR
Genes
BRS-73
BCT28
BCT37
BCT33
BCT38
BCT29
BRS-129
BCT31
BRS-124
BCT04
BCT12
BCT32
BCT36
BCT39
BSO19B
BCT14
BCT24
OP-535
BCT34
BCT16
BCT22
BCT26
BCT35
BCT30
BCT40
BSO36
BSO37
DHF168
BSO28
BSO30
BSO29
BSO32N
Samples

Figure S2

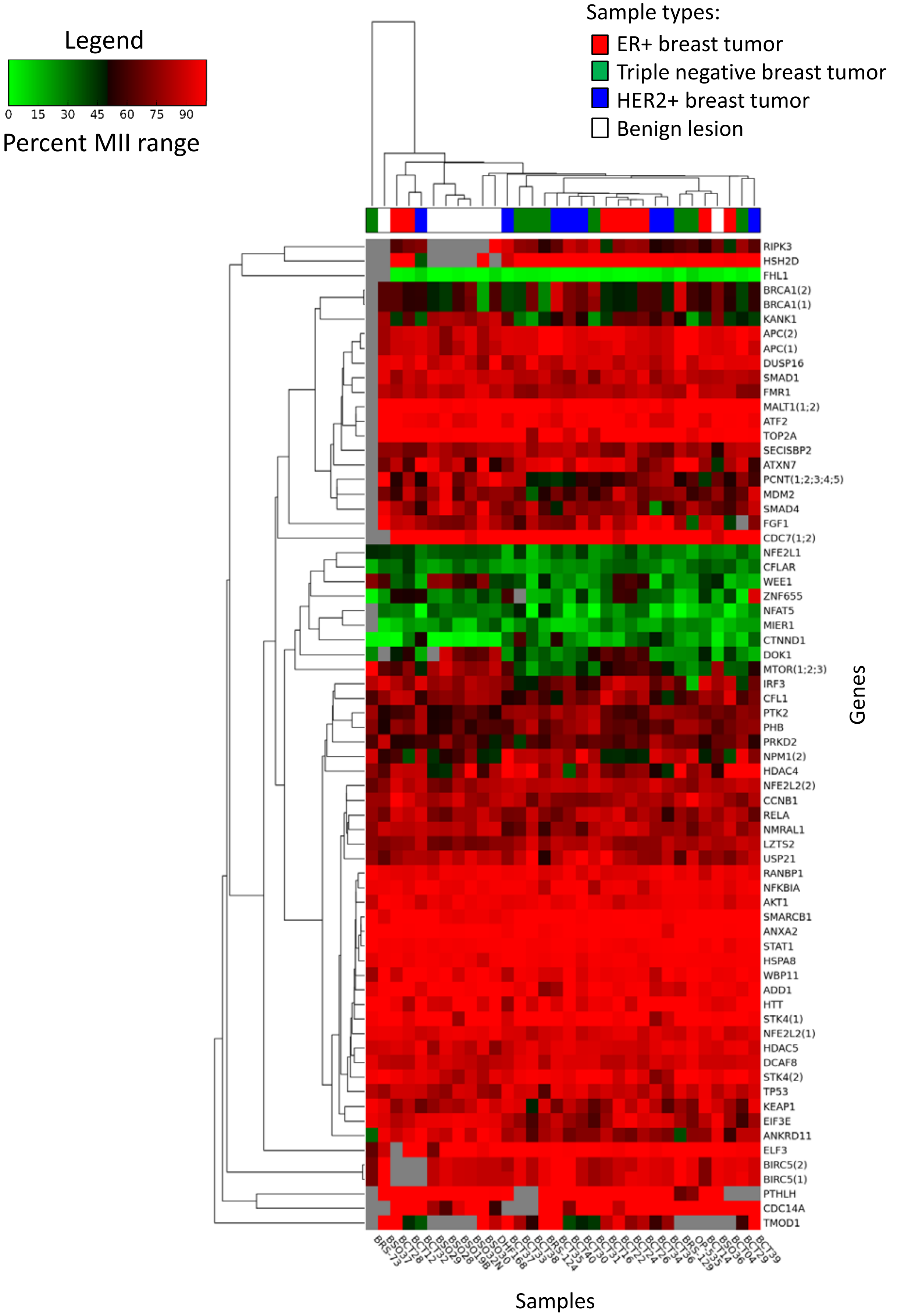

Legend
0 15 30 45 60 75 90
Percent MII range
Sample types:
ER+ breast tumor
Triple negative breast tumor
HER2+ breast tumor
Benign lesion
RIPK3
HSH2D
FHL1
BRCA1(2)
BRCA1(1)
KANK1
APC(2)
APC(1)
DUSP16
SMAD1
FMR1
MALT1(1;2)
ATF2
TOP2A
SECISBP2
ATXN7
PCNT(1;2;3;4;5)
MDM2
SMAD4
FGF1
CDC7(1;2)
NFE2L1
CFLAR
WEE1
ZNF655
NFAT5
MIER1
CTNND1
DOK1
MTOR(1;2;3)
IRF3
CFL1
PTK2
PHB
PRKD2
NPM1(2)
HDAC4
NFE2L2(2)
CCNB1
RELA
NMRAL1
LZTS2
USP21
RANBP1
NFKBIA
AKT1
SMARCB1
ANXA2
STAT1
HSPA8
WBP11
ADD1
HTT
STK4(1)
NFE2L2(1)
HDAC5
DCAF8
STK4(2)
TP53
KEAP1
EIF3E
ANKRD11
ELF3
BIRC5(2)
BIRC5(1)
PTHLH
CDC14A
TMOD1
Genes
BRS-73
BSO37
BCT28
BCT12
BCT32
BSO29
BSO28
BSO19B
BSO32N
BSO30
DHF168
BCT37
BCT33
BCT38
BRS-124
BCT35
BCT40
BCT30
BCT31
BCT16
BCT22
BCT24
BCT26
BCT34
BCT36
BRS-129
OP-535
BCT14
BSO36
BCT04
BCT29
BCT39
Samples

Figure S3
